# Molecular clustering of genes related to the atopic syndrome: Towards a more tailored approach and personalized medicine?

**DOI:** 10.1186/s13601-019-0273-8

**Published:** 2019-07-10

**Authors:** Jill de Wit, Rogier T. A. van Wijck, Virgil A. S. H. Dalm, Kristen L. Snyder, Joan E. E. Totté, Suzanne G. M. A. Pasmans, Peter J. van der Spek, the Academic Center of Excellence (ACE) workgroups Allergic Diseases and Rare Immunological Disease Centre (RIDC)

**Affiliations:** 1 Department of Dermatology, Erasmus MC University Medical Center, Dr. Molewaterplein 40, 3015 GD Rotterdam, The Netherlands; 2 Department of Internal Medicine, Division of Clinical Immunology, Erasmus MC University Medical Center, Dr. Molewaterplein 40, 3015 GD Rotterdam, The Netherlands; 3 Department of Immunology, Erasmus MC University Medical Center, Dr. Molewaterplein 40, 3015 GD Rotterdam, The Netherlands; 4 Department of Pathology, Division of Bioinformatics, Erasmus MC University Medical Center, Dr. Molewaterplein 40, 3015 GD Rotterdam, The Netherlands; 5 Department of Pediatric Dermatology, Sophia Children’s Hospital, Erasmus MC University Medical Center, Dr. Molewaterplein 40, 3015 GD Rotterdam, The Netherlands

**Keywords:** Atopy, Endotypes, Personalized medicine, Primary immunodeficiency disease

## Abstract

**Background:**

The atopic syndrome consists of heterogeneous manifestations, in which multiple associated genetic loci have recently been identified. It is hypothesized that immune dysregulation plays a role in the pathogenesis. In primary immunodeficiency diseases (PIDs), which are often monogenic immunodysregulation disorders, the atopic syndrome is a frequently occurring comorbidity. Based on the genetic defects in PIDs, novel gene/pathway-targeted therapies have been evaluated, which could be relevant in the atopic syndrome as well. Therefore, we aimed to define subclasses within the atopic syndrome based on the expression profiles of immune cell lineages of healthy mice.

**Methods:**

Overlap between known atopy-related genes as described in the Human Gene Mutation Database and disease-causing genes of monogenic PIDs was evaluated. Clusters of atopy-related genes were based on the overlap in their co-expressed genes using the gene expression profiles of immune cell lineages of healthy mice from the Immunological Genome Project. We analyzed pathways involved in the atopic syndrome using Ingenuity Pathway Analysis.

**Results:**

Twenty-two (5.3%) genes were overlapping between the atopy-related genes (n = 160) and PID-related genes (n = 278). We identified seven distinct clusters of atopy-related genes. Functional pathway analysis of all atopy-related genes showed relevance of T helper cell-mediated pathways.

**Conclusions:**

This study shows a model to define clusters within the atopic syndrome based on gene expression profiles of immune cell lineages. Our results support the hypothesis that both genetic mechanisms and immune dysregulation play a role in the pathogenesis. It also opens up the possibility for novel therapeutic targets and a more tailored approach towards personalized medicine.

**Electronic supplementary material:**

The online version of this article (10.1186/s13601-019-0273-8) contains supplementary material, which is available to authorized users.

## Background

Atopy is the genetic predilection to produce specific immunoglobulin (Ig) E following exposure to allergens. This predisposition results in the development of atopic dermatitis (AD), food allergy (FA), asthma and allergic rhinitis (AR): the atopic syndrome [1]. The worldwide prevalence of these manifestations in children varies between 15–20%, 1–10%, 3–29% and 9–15%, respectively, and in adults from 1–3%, 3–4%, 2–12% and 7–42%, respectively [2–6]. Atopic manifestations share a common mechanism involving allergen-specific IgE, which triggers the release of inflammatory mediators, like histamine, in the skin, gastrointestinal tract, lungs and nose. The course of these manifestations over time is characterized by the atopic march, generally starting with AD in infancy and followed by FA, asthma and AR later in childhood [7]. However, it is known that the atopic march not always follows the classic sequence and may occur at any age [8, 9]. Furthermore, not all atopic patients will develop the complete spectrum of atopic manifestations [7]. Despite its heterogeneous presentation, patients with atopic manifestations are mostly uniformly treated with topical or systemic immunosuppressive agents and/or antihistamines resulting in varying therapeutic responses as well [10–13].

Subgroups of the atopic phenotype, termed endotypes, are possibly responsible for the differences in disease manifestations and treatment responses. These endotypes are the result of variations in physiologic, biologic, immunologic and/or genetic mechanisms [14]. Various genetic loci associated with both inflammation and multiple atopic manifestations have been identified in recent years based on genome-wide association studies (GWAS), showing common genetic mechanisms involved in the atopic syndrome [15–24]. Nevertheless, the genetics of the atopic syndrome remain complicated for different reasons. For example, gene polymorphisms in different genes might cause the atopic syndrome independent of each other, and bearing a predisposing gene polymorphism does not necessarily result in development of the atopic syndrome [24]. The genetic complexity in the atopic syndrome possibly results in its heterogeneous clinical phenotype. Defining the endotypic profile of atopic patients in more detail contributes to determination of more homogeneous subclasses of patients. Subclasses are currently defined based on clinical and immunological characteristics, like the type of immune response involved [25]. However, stratification of atopic patients based on their genetic defect or polymorphism linked to their expression profile of immune cell lineages has not yet been investigated. This endotyping approach could be of interest as immune dysregulation may play an important role in the pathogenesis of the atopic syndrome. Interestingly, the atopic syndrome is a prevalent comorbidity in primary immunodeficiency diseases (PIDs), for example in hyper IgE syndrome (HIES), Comèl Netherton syndrome and immunodysregulation polyendocrinopathy enteropathy X-linked (IPEX) syndrome, which suggests that the atopic syndrome could be caused by a genetic defect in pathways that are also involved in these monogenic PIDs [26, 27]. This is supported by the hypothesis of autoallergy, in which atopy seems to stand at the boundary between allergy and auto-immunity, given the presence of IgE antibodies against self-proteins [28–30].

Several gene-targeted and/or pathway-targeted treatment strategies for PIDs have recently been under clinical evaluation, which could be of clinical benefit in the atopic syndrome as well. Identification of genetic pathways for these targeted and personalized treatment modalities is therefore essential.

We hypothesize that subclasses within the atopic syndrome exist based on genes that act in the same molecular pathway. Additionally, genetic defects in pathways that cause PID might also be involved in the atopic syndrome.

Therefore, the aim of this study is to define subclasses within the atopic syndrome via molecular clustering of atopy-related genes based on their expression profiles of immune cell lineages. We first evaluated the overlap between atopy-related genes and monogenic PID genes. Secondly, we clustered the atopy-related genes based on their expression profile of immune cell lineages of healthy mice. Finally, we analyzed the pathways in which the atopy-related genes are involved.

## Methods

### Data collection and content: overlap atopy/PID genes

We obtained a complete list of all mutated genes responsible for atopic manifestations by performing a comprehensive search in the Human Gene Mutation Database (HGMD, HGMD^®^ Professional, https://portal.biobase-international.com) up to August 21th 2018 [31]. Genes were searched using the phenotype terms “atopy”, “increased IgE”, “atopic dermatitis”, “eczema”, “food allergy”, “allergy”, “asthma” and “allergic rhinitis”. Atopy-related genes and the number of mutations per gene were extracted. Additionally, disease-causing genes of monogenic PIDs were obtained from the phenotypic classification for PIDs of the International Union of Immunological Societies (IUIS) [32]. We performed a cross check on atopy-related mutations in PID genes using HGMD. Overlapping genes between both the HGMD and PID lists were identified to select atopy-related with a defect in the same gene as a PID.

### Clustering and visualization of atopy-related genes

The atopy-related genes were clustered to identify more homogeneous subclasses of the atopic syndrome. Clusters were made based on their gene expression profiles of immune cell lineages. Therefore, gene expression data from the Immunological Genome Project (ImmGen, http://www.immgen.org) was downloaded from the Genome Expression Omnibus (GEO) database accession number GSE15907 and GSE37448. The ImmGen datasets comprise the gene expression of a large amount of immune cell lineages (both hematopoietic and mesenchymal), that were grouped into 12 cell-populations. Currently, there is limited data on the gene expression signatures of human immune cell types. Therefore, immune cell lineages of healthy mice were used, which might give insights in atopic processes also applicable in human. All atopy-related genes selected via the HGMD query were searched in the ImmGen dataset. The top 40 co-expressed genes in mice were extracted per atopy-related gene. These genes are of biological interest as co-expressed genes are controlled by the same transcriptional regulatory program, functionally related, or members of the same pathway or protein complex as our atopy-related genes of interest [33]. We overlaid the co-expressed genes to identify genes that occurred in the top 40 lists of multiple atopy-related genes. Based on the overlap in co-expressed genes, indicating the degree of similar expression of atopy-related genes, the atopy-related genes were clustered in an unsupervised manner. Accordingly, it is likely that the clustered atopy-related genes act in the same molecular pathway. The clusters were visualized by constructing a correlation network plot using the “qgraph” package in RStudio version 3.4.1 [34]. The lines between the genes were weighted and only correlations with a minimum correlation coefficient of 0.65, indicating a strong (positive) relationship, were visualized. If the top 40 list of an atopy-related gene did not contain a single overlapping gene, this atopy-related gene was labeled as an unclustered “bin” gene.

To visualize the gene expression profiles of the clusters, a heat map of the gene expression per cell lineage was constructed. Therefore, gene expression data were imported into Omniviz software version 6.1.13.0. Using Omniviz, the geometric mean of each probeset was calculated and transcriptomic data was log2 transformed to normalize the data. Changes in gene expression were constituted by deviations from the geometric mean to visualize whether genes of immune cell lineages were higher or lower expressed. These deviations are visualized in a heat map by a gradient from red (high expression) to blue (low expression) and ordered per cluster.

### Functional pathway analysis

We validated whether the extracted genes from HGMD were atopy-related through analysis of the pathways containing these atopy-related genes. As the separate clusters included small numbers of genes, all clustered atopy-related genes from HGMD with and without unclustered “bin” genes were analyzed using Ingenuity Pathway Analysis (IPA, Qiagen©) software [35]. The most important pathways, in which the atopy-related genes were involved, were extracted from IPA. The pathways were ranked according to their *p* value (-log transformed) and the ratio of the atopy-related genes found in each pathway over the total number of molecules in that pathway, indicating the significance of the association between the atopy-related genes and the identified pathways. The *p* value was calculated using a Fisher’s exact test to determine the probability that the association between the atopy-related genes and the pathways is explained by a random chance alone. A –log (*p* value) equal to or greater than 1.3, corresponding to a *p* value of 0.05, was considered statistically significant.

## Results

### Content of data

**Fig. 1:**
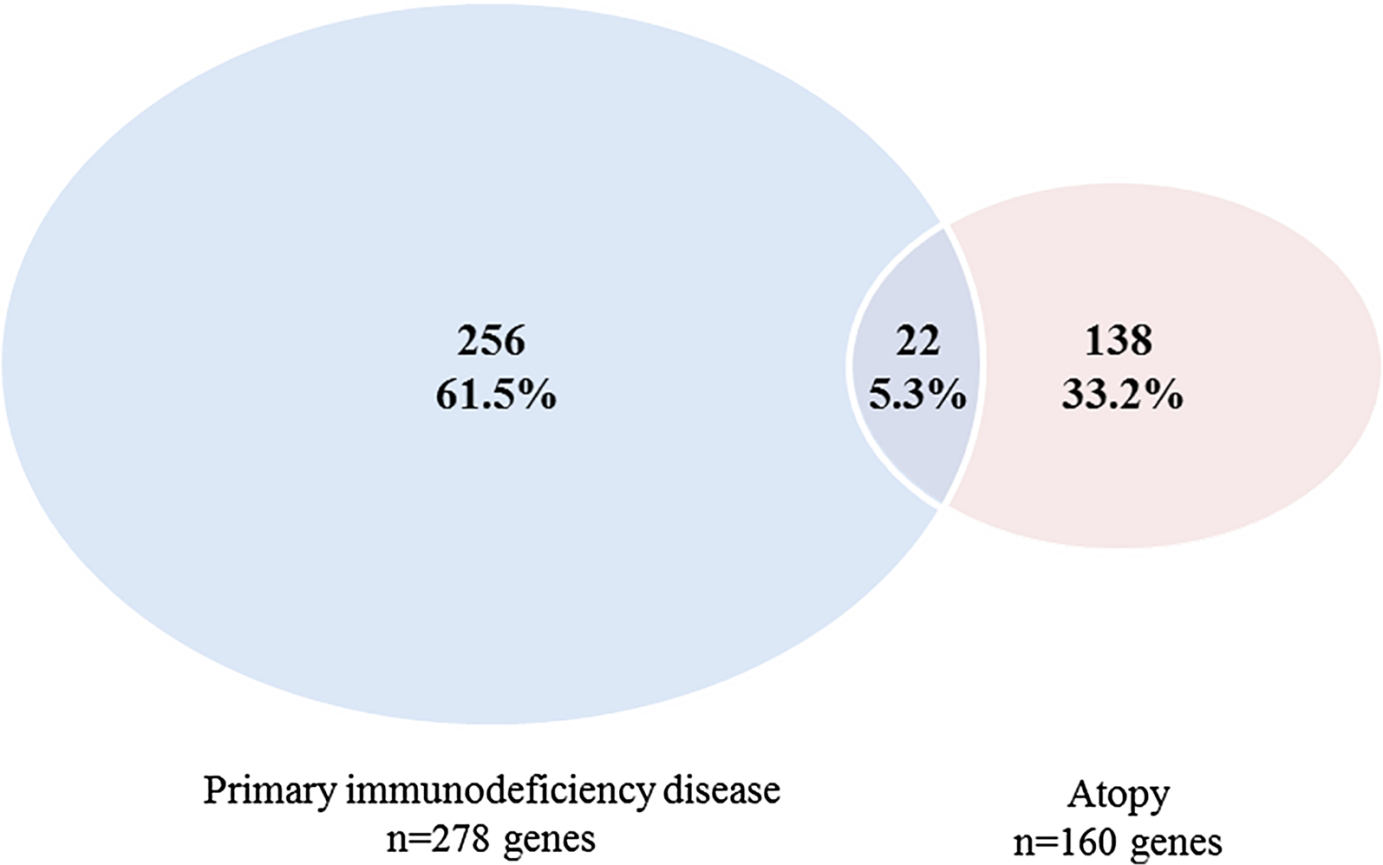
Venn diagram illustrating the overlap of the disease causing genes of monogenic primary immunodeficiency diseases and the atopy-related genes identified in the Human Gene Mutation Database

The search in HGMD on atopic manifestations retrieved 159 atopy-related genes known in human (Additional file 1: Table S1). Based on the overview of the IUIS, 278 disease-causing genes of monogenic PIDs were obtained [36]. During the cross-check on atopy-related mutations in PID genes, *TRAF3IP2* was identified of which mutations were described that might result in an eczema phenotype. This gene did not appear in the search results of HGMD and was therefore added to the list of atopy-related genes, resulting in a total of 160 genes for further analysis. The top three genes with the highest number of atopy-related mutations included *STAT3* (n = 107), *FLG* (n = 62) and *DOCK8* (n = 45). Other genes had six or less atopy-related mutations per gene (Additional file 1: Table S1). Twenty-two (5.3%) genes of the atopy (n = 160) and PID (n = 278) lists were overlapping, including *ARPC1B*, *BTK*, *CASP8*, *CFTR*, *CTLA4*, *DOCK8*, *ICOS*, *IL10*, *IL12B*, *IL12RB1*, *IL17F*, *IL21*, *IL21R*, *IL7R*, *ITK*, *ORAI1*, *PGM3*, *SPINK5*, *STAT3*, *TNFRSF13B*, *TRAF3IP2* and *TYK2* (Fig. 1 and Additional file 1: Table S1).

### Clustering of genes

**Fig. 2:**
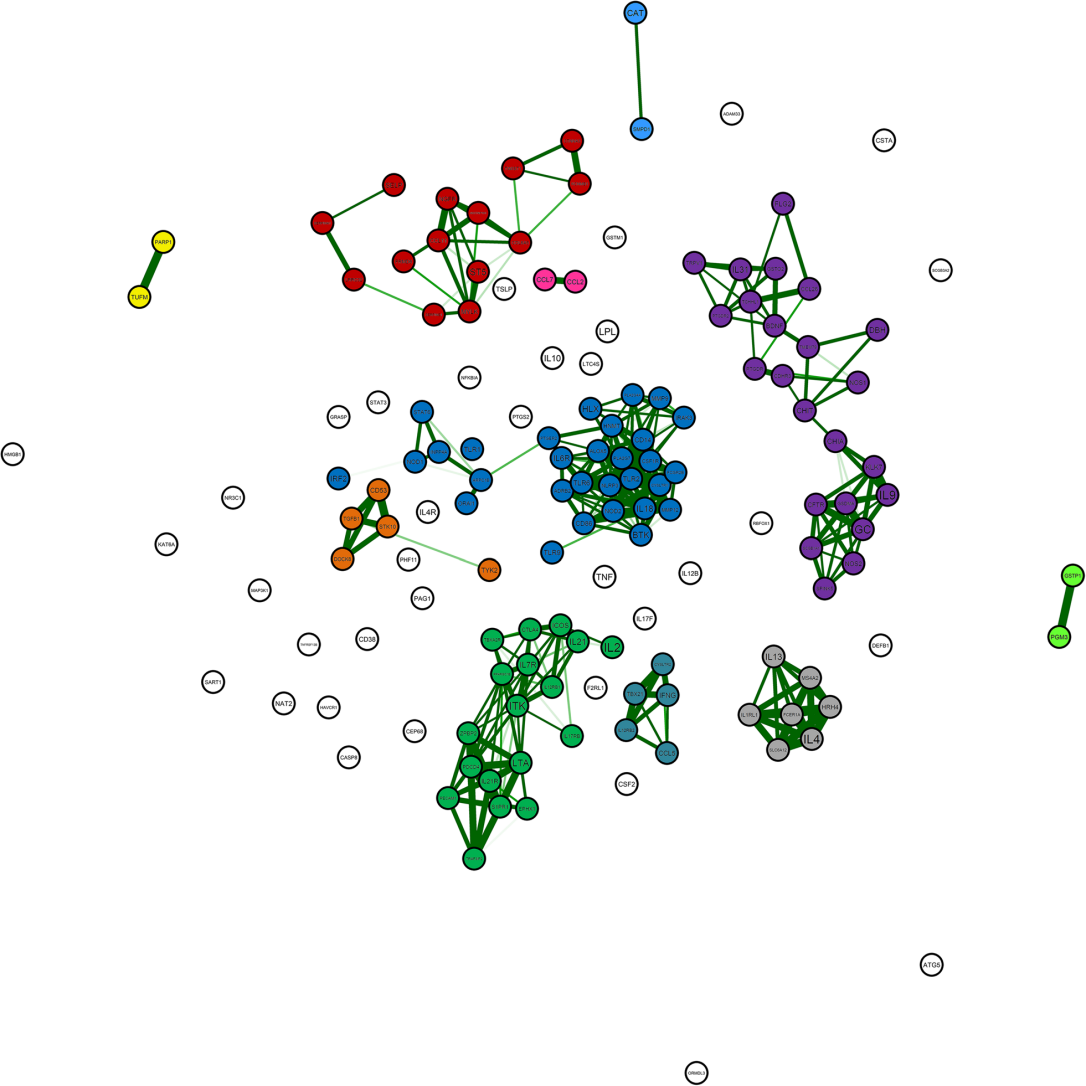
Genetic correlation network plot of atopy-related gene clusters. The line width between the atopy-related genes indicate the overlay in the top 40 co-expressed gene lists per atopy-related gene and is proportional to the strength of correlation within the clusters. Only those with correlation coefficients > 0.65 are visualized

**Fig. 3:**
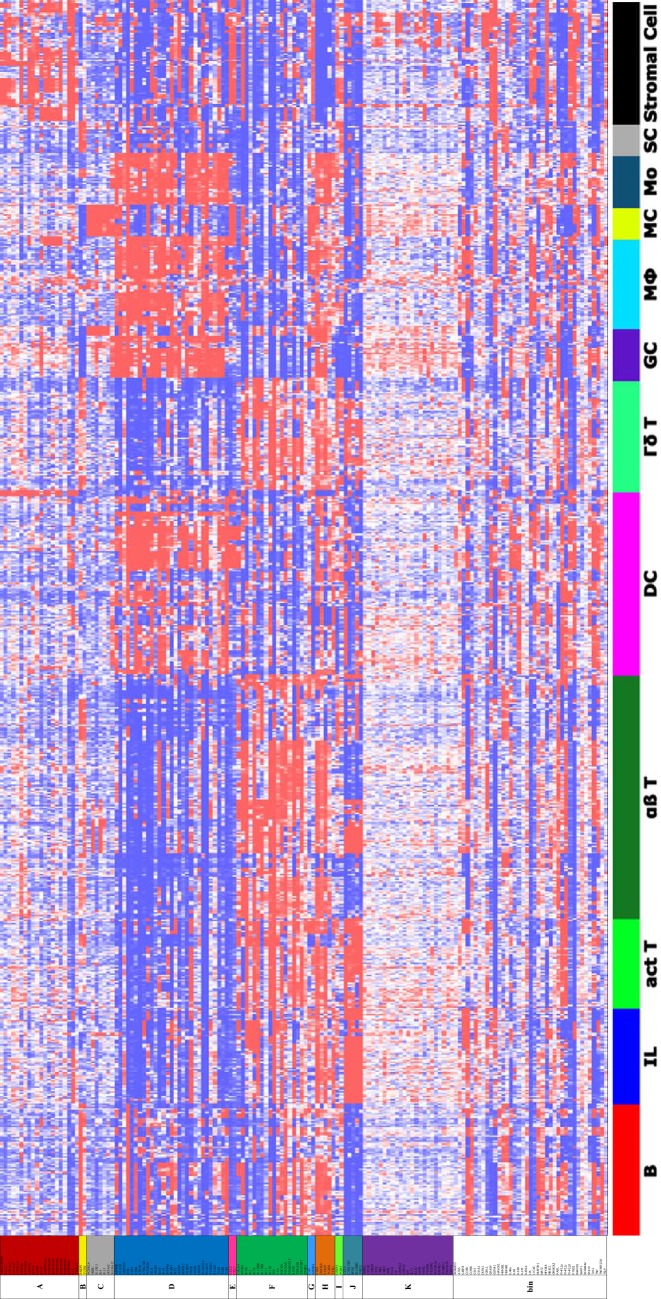
Heat map representing the atopy-related gene expression across the immune cell lineage of healthy mice ordered according to the identified clusters within the atopic syndrome. Data on the expression of atopy-related genes across the immune cell lineages was constructed using the Omniviz software, in which changes in gene expression were visualized by a gradient from red (high expression) to blue (low expression). Genes were alphabetically ordered according to the identified genetic cluster. Thirty-seven non-correlated genes remained as “bin”. Abbreviations: B, B lymphocyte; IL, innate lymphocyte; act T, activated T lymphocyte; αβ T, αβ T lymphocyte; DC, dendritic cell; Γδ T, Γδ T lymphocyte, GC, granulocyte; MΦ, macrophage; MC, mast cell; Mo, monocyte; SC, stem cell

Fifteen (9.4%) of the 160 atopy-related genes were not expressed in the mouse immune system, of which immune cell lineages were used in the ImmGen dataset, and were therefore excluded from further analysis. As some genes had multiple transcripts and appeared more than once in the gene expression dataset, the complete list for clustering resulted in 153 probes. Eleven clusters were identified, of which seven clusters included five or more genes (clusters A, C, D, F, H, J and K), and 37 non-correlated genes remained as “bin” (Figs. 2 and 3, Additional file 1: Table S1). Based on the gene expression profiles, we identified one pair of anti-correlated clusters (clusters D and F), i.e. opposite expression profiles between clusters D and F (Fig. 3). The 22 overlapping genes between the atopy-related genes and monogenic PID genes were localized in two of the seven atopy-related gene clusters, including cluster F (n = 8) and cluster D (n = 3) (Additional file 1: Table S1).

### Functional pathway analysis

Functional pathway analysis in IPA of the atopy-related genes both with and without taking unclustered “bin” genes into account resulted in T helper (Th) cell-mediated pathways. Based on all atopy-related genes (n = 160), this included the specific pathways “T helper cell differentiation”, “Th1 and Th2 activation pathway”, and “Th2 pathway”, in which respectively 22, 28 and 24 atopy-related genes were involved (Additional file 2: Table S2a and S2b). Additionally, pathway analysis of the clustered atopy-related genes only (n = 108) resulted in the specific pathways “Th1 and Th2 activation pathway” (n = 22 genes), “T-helper cell differentiation” (n = 16 genes), and “Th2 pathway” (n = 19 genes) (Additional file 2: Table S2c and S2d).

## Discussion

This is the first study that describes clusters in the clinically heterogeneous phenotype of the atopic syndrome based on gene expression profiles of immune cell lineages of healthy mice. The overlap between atopy-related genes (n = 160) and monogenic PID genes (n = 278) was limited to 22 (5.3%) genes. We identified seven distinct clusters within the atopic syndrome based on the expression profiles of atopy-related genes. Functional pathway analysis of all known atopy-related genes resulted in identification of Th cell-mediated processes underlying the atopic syndrome.

The atopic syndrome is a prevalent comorbidity in a number of PIDs, suggesting that the atopic syndrome can be a symptom of PIDs and that immune dysregulation plays a role in the pathogenesis. Interestingly, the number of overlapping genes in this study was limited (5.3%) and did not belong to one PID category according to the IUIS phenotypic classification or immunologic component [32]. Nonetheless, the overlapping genes were bundled in just two of the seven atopy-related gene clusters (cluster D and F), which suggests that these endotypes of the atopic syndrome are associated with the predisposition to develop a PID. However, atopy-related mutations in these genes might differ from the disease-causing mutations of the PIDs.

Current literature reports nine PIDs to be possibly related to the atopic syndrome, including autosomal dominant HIES (AD-HIES; *STAT3*), autosomal recessive HIES (AR-HIES; *DOCK8*), Comèl Netherton syndrome (*SPINK5*), hypogammaglobulinemia, selective IgA deficiency (SIgAD), IgM deficiency, IPEX (*FOXP3*), chronic granulomatous disease (CGD; *CYBA, CYBB, NCF1*, *NCF2* and *NCF4*), and phospholipase C gamma 2 (PLCG2) gene associated antibody deficiency and immune dysregulation (PLAID; *PLCG2*), and 28 additional genetic PID conditions [27, 37]. Only eight genes (*STAT3, DOCK8*, *SPINK5*, *FLG*, *ARPC1B*, *PGM3*, *ERBIN* and *TYK2*) were extracted from HGMD using the atopic phenotype search. Furthermore, only two of the 22 overlapping atopy-related and PID-related genes identified in this study were reported in literature to be involved in PIDs and the atopic syndrome [27]. The discrepancy between literature and HGMD could firstly be explained by the recent expansion of novel mutations derived from next generation sequencing (NGS). Secondly, the atopic manifestations in PIDs, as described in literature, might be an occasional finding and not related to the disease causing genes of PIDs. Thirdly, the heterogeneous course and presentation of the atopic syndrome may make it difficult to associate genetic mutations with atopic manifestations. Moreover, the infectious symptoms in PIDs might be a more prominent clinical feature than the atopic manifestations, which therefore could have resulted in a registration bias.

We found a low number of mutations in most atopy-related genes in human (six or less mutations in 157 of the 160 genes), suggesting that other phenomena contribute to the disease such as post-translational modifications. Alternatively, various genes that interact with environmental factors might be involved in the atopic syndrome, in which each gene contributes only to a small amount of the overall disease risk [38]. Furthermore, the differences between the clusters could indicate that immune regulation plays a role in the atopic syndrome next to underlying genetic mechanisms.

Strikingly, two of the identified clusters (D and F) have a completely opposite expression profile, both in lymphoid and myeloid cell lineages. An explanation for this phenomenon may be that both clusters share the same upstream regulator. Depending on a gain or loss of function mutation in this enhancer, the gene expression profile can be influenced by an agonist or antagonist of this regulator. By performing a functional pathway analysis of the atopy-related genes in only clusters D and F, we would explore the functional significance of these clusters. The analysis resulted in the pathways “T helper cell differentiation”, “TREM1 signaling” and “Th1 and Th2 activation pathway”, which is completely corresponding with the pathways involved in all atopy-related genes (data not shown). Therefore, we could unfortunately not differentiate between the functional significance of all atopy-related genes and those included in clusters D and F.

The identified Th cell-mediated pathway supports the hypothesis that changes in the immune system underlie and could be involved in the pathogenesis of atopy. In AD it has been previously described that acute skin lesions are characterized by Th2 cell infiltration with a shift towards predominantly Th1 cells in the chronic phase [39–41]. In addition, asthma was reported as a Th2 cell-mediated diseases driven by allergen exposure [42]. Moreover, patients with FA and AR are characterized by allergen-specific Th2 cell-mediated responses showing that the obtained Th cell-pathways involved in the atopic syndrome are in agreement with these of the individual atopic manifestations [43–45]. In most of our identified clusters (except clusters F, G, H, I and J) the atopy-related genes do not show increased expression in T lymphocytes (Fig. 3). Therefore, genes in these clusters might be expressed in immunologic cells that co-interact with T lymphocytes, including Th cells, or in cells that are progenitors of Th cells.

This study has some limitations. Firstly, we might have missed gene expression profiles of barrier cells as we could not include terms concerning the skin barrier in the phenotype search in HGMD. However, by using the terms “atopic dermatitis” and “eczema” we have identified important barrier genes, like *COL6A5*, *FLG* (subtypes), *FLG2*, and *KLK7*. Secondly, some discrepancies were found in the HGMD database. The genes from the atopic phenotype search did not completely overlap with the results from the search on atopy-related mutations per gene. Therefore, we identified atopic phenotypes per gene on the results of both searches. Thirdly, we clustered genes based on their expression profiles in the ImmGen dataset, which uses characterized immune cells of healthy mice. The gene expression profiles of immune cell lineages in healthy mice may not be identical to these in (atopic) human. This explains why we could not cluster all human atopy-related genes including *FLG*, which is an important atopy-related gene based on the number of atopy-related mutations (n = 62). Furthermore, the data from mice cannot directly be applied for subgrouping of the atopic syndrome in human. Therefore, large cohorts of patients with the atopy phenotype should be sequenced using NGS to investigate whether atopy clusters could be generated based on the gene expression profiles of immune cell lineages of atopic human. Identification of clusters of atopy-related genes by NGS potentially opens novel ways to select eligible patients for pharmaceutical studies and could predict therapeutic responses.

## Conclusions

This study shows a model, using data of healthy mice, to define clusters of the atopic syndrome based on gene expression profiles of immune cell lineages. We identified seven distinct clusters within the atopic syndrome in which Th cell-mediated pathways were most often involved. This supports the hypothesis that both genetic mechanisms and immune dysregulation have a role in the pathogenesis the atopic syndrome. It also opens up the possibility for identification of novel therapeutic targets towards a more tailored approach and personalized medicine.

## Additional files

**Additional file 1: Table S1.** Atopy-related genes from the Human Gene Mutation Database.

**Additional file 2: Table S2.** (a) Ingenuity Pathway Analysis – top three pathways of all atopy-related genes (n = 160); (b) Ingenuity Pathway Analysis - all atopy-related genes (n = 160); (c) Ingenuity Pathway Analysis – top 3 pathways of atopy-related genes without ‘bin’ genes (n = 108); (d) Ingenuity Pathway Analysis – atopy-related genes without ‘bin’ genes (n = 108).

## Data Availability

The HGMD dataset analyzed during the current study are available via https://portal.biobase-international.com/cgi-bin/portal/login.cgi?redirect_url=/hgmd/pro/start.php.

## Abbreviations

AD: atopic dermatitis
AD-HIES: autosomal dominant hyper IgE syndrome
AR: allergic rhinitis
AR-HIES: autosomal recessive hyper IgE syndrome
CGD: chronic granulomatous disease
FA: food allergy
GEO: Genome Expression Omnibus
GWAS: genome-wide association studies
HGMD: Human Gene Mutation Database
HIES: hyper IgE syndrome
ImmGen: Immunological Genome Project
IPA: Ingenuity Pathway Analysis
IUIS: International Union of Immunological Societies
Ig: immunoglobulin
IPEX: immunodysregulation polyendocrinopathy enteropathy X-linked
NGS: next generation sequencing
PID: primary immunodeficiency disease
PLAID: phospholipase C gamma 2 gene associated antibody deficiency and immune dysregulation
PLCG2: phospholipase C gamma 2
SIgAD: selective immunoglobulin A deficiency
Th: T helper

## References

[1] Tan RA, Corren J (2011). The relationship of rhinitis and asthma, sinusitis, food allergy, and eczema. Immunol Allergy Clin North Am.

[2] Asher MI, Montefort S, Bjorksten B, Lai CK, Strachan DP, Weiland SK, Williams H, Group IPTS (2006). Worldwide time trends in the prevalence of symptoms of asthma, allergic rhinoconjunctivitis, and eczema in childhood: ISAAC Phases One and Three repeat multicountry cross-sectional surveys. Lancet.

[3] Loh W, Tang M (2018). The epidemiology of food allergy in the global context. Int J Environ Res Public Health.

[4] Variations in the prevalence of respiratory symptoms (1996). self-reported asthma attacks, and use of asthma medication in the European Community Respiratory Health Survey (ECRHS). Eur Respir J.

[5] Izquierdo-Dominguez A, Valero AL, Mullol J (2013). Comparative analysis of allergic rhinitis in children and adults. Curr Allergy Asthma Rep.

[6] Nutten S (2015). Atopic dermatitis: global epidemiology and risk factors. Ann Nutr Metab.

[7] Dharmage SC, Lowe AJ, Matheson MC, Burgess JA, Allen KJ, Abramson MJ (2014). Atopic dermatitis and the atopic march revisited. Allergy.

[8] Barberio G, Pajno GB, Vita D, Caminiti L, Canonica GW, Passalacqua G (2008). Does a ‘reverse’ atopic march exist?. Allergy.

[9] Burgess JA, Dharmage SC, Byrnes GB, Matheson MC, Gurrin LC, Wharton CL, Johns DP, Abramson MJ, Hopper JL, Walters EH (2008). Childhood eczema and asthma incidence and persistence: a cohort study from childhood to middle age. J Allergy Clin Immunol.

[10] Dykewicz MS, Wallace DV, Baroody F, Bernstein J, Craig T, Finegold I, Huang F, Larenas-Linnemann D, Meltzer E, Steven G (2017). Treatment of seasonal allergic rhinitis: an evidence-based focused 2017 guideline update. Ann Allergy Asthma Immunol.

[11] Eichenfield LF, Tom WL, Berger TG, Krol A, Paller AS, Schwarzenberger K, Bergman JN, Chamlin SL, Cohen DE, Cooper KD, Cordoro KM, Davis DM, Feldman SR, Hanifin JM, Margolis DJ, Silverman RA, Simpson EL, Williams HC, Elmets CA, Block J, Harrod CG, Smith Begolka W, Sidbury R (2014). Guidelines of care for the management of atopic dermatitis: section 2. Management and treatment of atopic dermatitis with topical therapies. J Am Acad Dermatol.

[12] Sidbury R, Davis DM, Cohen DE, Cordoro KM, Berger TG, Bergman JN, Chamlin SL, Cooper KD, Feldman SR, Hanifin JM, Krol A, Margolis DJ, Paller AS, Schwarzenberger K, Silverman RA, Simpson EL, Tom WL, Williams HC, Elmets CA, Block J, Harrod CG, Begolka WS, Eichenfield LF (2014). Guidelines of care for the management of atopic dermatitis: section 3. Management and treatment with phototherapy and systemic agents. J Am Acad Dermatol.

[13] Boyce JA, Assa’ad A, Burks AW, Jones SM, Sampson HA, Wood RA, Plaut M, Cooper SF, Fenton MJ, Arshad SH (2010). Guidelines for the diagnosis and management of food allergy in the United States: report of the NIAID-sponsored expert panel. J Allergy Clin Immunol.

[14] Collins FS, Varmus H (2015). A new initiative on precision medicine. N Engl J Med.

[15] Paternoster L, Standl M, Waage J, Baurecht H, Hotze M, Strachan DP, Curtin JA, Bonnelykke K, Tian C, Takahashi A, Esparza-Gordillo J, Alves AC, Thyssen JP, denDekker HT, Ferreira MA, Altmaier E, Sleiman PM, Xiao FL, Gonzalez JR, Marenholz I, Kalb B, Yanes MP, Xu CJ, Carstensen L, Groen-Blokhuis MM, Venturini C, Pennell CE, Barton SJ, Levin AM, Curjuric I, Bustamante M, Kreiner-Moller E, Lockett GA, Bacelis J, Bunyavanich S, Myers RA, Matanovic A, Kumar A, Tung JY, Hirota T, Kubo M, McArdle WL, Henderson AJ, Kemp JP, Zheng J, Smith GD, Ruschendorf F, Bauerfeind A, Lee-Kirsch MA, Arnold A, Homuth G, Schmidt CO, Mangold E, Cichon S, Keil T, Rodriguez E, Peters A, Franke A, Lieb W, Novak N, Folster-Holst R, Horikoshi M, Pekkanen J, Sebert S, Husemoen LL, Grarup N, deJongste JC, Rivadeneira F, Hofman A, Jaddoe VW, Pasmans SG, Elbert NJ, Uitterlinden AG, Marks GB, Thompson PJ, Matheson MC, Robertson CF, Ried JS, Li J, Zuo XB, Zheng XD, Yin XY, Sun LD, McAleer MA, O’Regan GM, Fahy CM, Campbell LE, Macek M, Kurek M, Hu D, Eng C, Postma DS, Feenstra B, Geller F, Hottenga JJ, Middeldorp CM, Hysi P, Bataille V, Spector T, Tiesler CM, Thiering E, Pahukasahasram B, Yang JJ, Imboden M, Huntsman S, Vilor-Tejedor N, Relton CL, Myhre R, Nystad W, Custovic A, Weiss ST, Meyers DA, Soderhall C, Melen E, Ober C, Raby BA, Simpson A, Jacobsson B, Holloway JW, Bisgaard H, Sunyer J, Hensch NMP, Williams LK, Godfrey KM, Wang CA, Boomsma DI, Melbye M, Koppelman GH, Jarvis D, McLean WI, Irvine AD, Zhang XJ, Hakonarson H, Gieger C, Burchard EG, Martin NG, Duijts L, Linneberg A, Jarvelin MR, Noethen MM, Lau S, Hubner N, Lee YA, Tamari M, Hinds DA, Glass D, Brown SJ, Heinrich J, Evans DM, Weidinger S, AustralianAsthma Genetics Consortium (2015). Multi-ancestry genome-wide association study of 21,000 cases and 95,000 controls identifies new risk loci for atopic dermatitis. Nat Genet.

[16] Hinds DA, McMahon G, Kiefer AK, Do CB, Eriksson N, Evans DM, St Pourcain B, Ring SM, Mountain JL, Francke U (2013). A genome-wide association meta-analysis of self-reported allergy identifies shared and allergy-specific susceptibility loci. Nat Genet.

[17] Ferreira MAR, Matheson MC, Duffy DL, Marks GB, Hui J, Le Souëf P, Danoy P, Baltic S, Nyholt DR, Jenkins M (2011). Identification of IL6R and chromosome 11q13. 5 as risk loci for asthma. The Lancet.

[18] Himes BE, Hunninghake GM, Baurley JW, Rafaels NM, Sleiman P, Strachan DP, Wilk JB, Willis-Owen SAG, Klanderman B, Lasky-Su J (2009). Genome-wide association analysis identifies PDE4D as an asthma-susceptibility gene. Am J Hum Genet.

[19] Noguchi E, Sakamoto H, Hirota T, Ochiai K, Imoto Y, Sakashita M, Kurosaka F, Akasawa A, Yoshihara S, Kanno N (2011). Genome-wide association study identifies HLA-DP as a susceptibility gene for pediatric asthma in Asian populations. PLoS Genet.

[20] Moffatt MF, Gut IG, Demenais F, Strachan DP, Bouzigon E, Heath S, Von Mutius E, Farrall M, Lathrop M, Cookson WOCM (2010). A large-scale, consortium-based genomewide association study of asthma. N Engl J Med.

[21] Sleiman PMA, Flory J, Imielinski M, Bradfield JP, Annaiah K, Willis-Owen SAG, Wang K, Rafaels NM, Michel S, Bonnelykke K (2010). Variants of DENND1B associated with asthma in children. N Engl J Med.

[22] Hirota T, Takahashi A, Kubo M, Tsunoda T, Tomita K, Doi S, Fujita K, Miyatake A, Enomoto T, Miyagawa T (2011). Genome-wide association study identifies three new susceptibility loci for adult asthma in the Japanese population. Nat Genet.

[23] Bønnelykke K, Matheson MC, Pers TH, Granell R, Strachan DP, Alves AC, Linneberg A, Curtin JA, Warrington NM, Standl M (2013). Meta-analysis of genome-wide association studies identifies ten loci influencing allergic sensitization. Nat Genet.

[24] Heinzmann A, Deichmann KA (2001). Genes for atopy and asthma. Curr Opin Allergy Clin Immunol.

[25] Muraro A, Lemanske RF, Hellings PW, Akdis CA, Bieber T, Casale TB, Jutel M, Ong PY, Poulsen LK, Schmid-Grendelmeier P, Simon HU, Seys SF, Agache I (2016). Precision medicine in patients with allergic diseases: airway diseases and atopic dermatitis-PRACTALL document of the European Academy of Allergy and Clinical Immunology and the American Academy of Allergy, Asthma & Immunology. J Allergy Clin Immunol.

[26] Pichard DC, Freeman AF, Cowen EW (2015). Primary immunodeficiency update: Part I. Syndromes associated with eczematous dermatitis. J Am Acad Dermatol.

[27] de Wit J, Brada RJK, van Veldhuizen J, Dalm VASH, Pasmans SGMA (2019). Skin disorders are prominent features in primary immunodeficiency diseases: a systematic overview of current data. Allergy.

[28] Bieber T (2008). Atopic dermatitis. N Engl J Med.

[29] Mittermann I, Aichberger KJ, Bunder R, Mothes N, Renz H, Valenta R (2004). Autoimmunity and atopic dermatitis. Curr Opin Allergy Clin Immunol.

[30] Aichberger KJ, Mittermann I, Reininger R, Seiberler S, Swoboda I, Spitzauer S, Kopp T, Stingl G, Sperr WR, Valent P, Repa A, Bohle B, Kraft D, Valenta R (2005). Hom s 4, an IgE-reactive autoantigen belonging to a new subfamily of calcium-binding proteins, can induce Th cell type 1-mediated autoreactivity. J Immunol.

[31] Stenson PD, Mort M, Ball EV, Shaw K, Phillips A, Cooper DN (2014). The Human Gene Mutation Database: building a comprehensive mutation repository for clinical and molecular genetics, diagnostic testing and personalized genomic medicine. Hum Genet.

[32] Bousfiha A, Jeddane L, Picard C, Ailal F, Bobby Gaspar H, Al-Herz W, Chatila T, Crow YJ, Cunningham-Rundles C, Etzioni A, Franco JL, Holland SM, Klein C, Morio T, Ochs HD, Oksenhendler E, Puck J, Tang MLK, Tangye SG, Torgerson TR, Casanova JL, Sullivan KE (2018). The 2017 IUIS phenotypic classification for primary immunodeficiencies. J Clin Immunol.

[33] Weirauch MT (2011). Gene coexpression networks for the analysis of DNA microarray data. Appl Stat Netw Biol Methods Syst Biol.

[34] Epskamp S, Costantini G, Haslbeck J, Cramer AOJ, Waldorp LJ, Schmittmann VD, Borsboom D, The qgraph package. Graph plotting methods, psychometric data visualization and graphical model estimation. https://cran.r-project.org/web/packages/qgraph/qgraph.pdf. Accessed June 2019.

[35] Kramer A, Green J, Pollard J, Tugendreich S (2014). Causal analysis approaches in ingenuity pathway analysis. Bioinformatics.

[36] Picard C, Bobby Gaspar H, Al-Herz W, Bousfiha A, Casanova JL, Chatila T, Crow YJ, Cunningham-Rundles C, Etzioni A, Franco JL, Holland SM, Klein C, Morio T, Ochs HD, Oksenhendler E, Puck J, Tang MLK, Tangye SG, Torgerson TR, Sullivan KE (2018). International union of immunological societies: 2017 primary immunodeficiency diseases committee report on inborn errors of immunity. J Clin Immunol.

[37] Ponsford MJ, Klocperk A, Pulvirenti F, Dalm V, Milota T, Cinetto F, Chovancova Z, Rial MJ, Sediva A, Litzman J, Agostini C, van Hagen M, Quinti I, Jolles S (2018). Hyper-IgE in the allergy clinic–when is it primary immunodeficiency?. Allergy.

[38] Geddes L. Huge brain study uncovers ‘buried’ genetic networks linked to mental illness. Nature. 2018. https://www.nature.com/articles/d41586-018-07750-x. Accessed June 2019.

[39] Biedermann T, Skabytska Y, Kaesler S, Volz T (2015). Regulation of T cell immunity in atopic dermatitis by microbes: the Yin and Yang of cutaneous inflammation. Front Immunol.

[40] Kaesler S, Volz T, Skabytska Y, Koberle M, Hein U, Chen KM, Guenova E, Wolbing F, Rocken M, Biedermann T (2014). Toll-like receptor 2 ligands promote chronic atopic dermatitis through IL-4-mediated suppression of IL-10. J Allergy Clin Immunol.

[41] Niebuhr M, Baumert K, Heratizadeh A, Satzger I, Werfel T (2014). Impaired NLRP3 inflammasome expression and function in atopic dermatitis due to Th2 milieu. Allergy.

[42] Lloyd CM, Saglani S (2013). T cells in asthma: influences of genetics, environment, and T-cell plasticity. J Allergy Clin Immunol.

[43] Turcanu V, Maleki SJ, Lack G (2003). Characterization of lymphocyte responses to peanuts in normal children, peanut-allergic children, and allergic children who acquired tolerance to peanuts. J Clin Invest.

[44] Flinterman AE, Pasmans SG, den Hartog Jager CF, Hoekstra MO, Bruijnzeel-Koomen CA, Knol EF, van Hoffen E (2010). T cell responses to major peanut allergens in children with and without peanut allergy. Clin Exp Allergy.

[45] Rondon C, Campo P, Togias A, Fokkens WJ, Durham SR, Powe DG, Mullol J, Blanca M (2012). Local allergic rhinitis: concept, pathophysiology, and management. J Allergy Clin Immunol.

