# Supplementary material for: Molecular clustering of genes related to the atopic syndrome: Towards a more tailored approach and personalized medicine?
Source: Clin Transl Allergy. 2019 Jul 10;9:34. doi: 10.1186/s13601-019-0273-8 (PMC6617681; doi:10.1186/s13601-019-0273-8)
Supplement: Supplementary file 1 — Additional file 1: Table S1. Atopy-related genes from the Human Gene Mutation Database. [file 13601_2019_273_MOESM1_ESM.pdf]

Additional File 1: Table S1. Atopy-related genes from the Human Gene Mutation Database

Gene symbol	Gene description	Atopic symptom involved	Number of atopy mutations	Cluster*
ADCYAP1R1	Adenylate Cyclase Activating Polypeptide 1 Receptor Type I	Asthma	NA	A
CCL11	C-C Motif Chemokine Ligand 11	Asthma	NA	A
COL6A5	Collagen Type VI Alpha 5 Chain	Dermatitis	4	A
CTNNA3	Catenin Alpha 3	Food allergy	2	A
FRMD6	FERM Domain Containing 6	Asthma	1	A
KCNMB1	Potassium Calcium-Activated Channel Subfamily M Regulatory Beta Subunit 1	Asthma	1	A
LRRC32	Leucine Rich Repeat Containing 32	Dermatitis	6	A
MYLK	Myosin Light Chain Kinase	Asthma	1	A
NGFR	Nerve Growth Factor Receptor	Asthma	NA	A
SELP	Selectin P	Atopy	1	A
SERPINA1	Serpin Family A Member 1	Asthma	1	A
SERPINE1	Serpin Family E Member 1	Asthma	NA	A
ST5	Suppression Of Tumorigenicity 5	Asthma	1	A
TWIST1	Twist Family BHLH Transcription Factor 1	IgE	NA	A
PARP1	Poly(ADP-Ribose) Polymerase 1	Asthma	NA	B
TUFM	Tu Translation Elongation Factor, Mitochondrial	Asthma	1	B
FCER1A	Fc Fragment Of IgE Receptor Ia	Dermatitis	1	C
HRH4	Histamine Receptor H4	Dermatitis	NA	C
IL1RL1	Interleukin 1 Receptor Like 1	Dermatitis	2	C
IL4	Interleukin 4	IgE<br>Asthma	3	C
IL13	Interleukin 13	Atopy<br>IgE<br>Asthma<br>Rhinitis	2	C
MS4A2	Membrane Spanning 4-Domains A2	Atopy<br>IgE<br>Asthma<br>Rhinitis	5	C
SLC6A12	Solute Carrier Family 6 Member 12	Asthma	1	C
ADRB2	Adrenoceptor Beta 2	Dermatitis<br>Asthma	2	D
ALOX5	Arachidonate 5-Lipoxygenase	Asthma	1	D
ARPC1B†	Actin Related Protein 2/3 Complex Subunit 1B	Allergy	1	D
BTK†	Bruton Tyrosine Kinase	Asthma	1	D
CD14	Cluster of Differentiation 14 Molecule	Asthma<br>Rhinitis	NA	D
CD86	Cluster of Differentiation 86 Molecule	Asthma	1	D
CSF1R	Colony Stimulating Factor 1 Receptor	Asthma	1	D
CYSLTR1	Cysteinyl Leukotriene Receptor 1	Atopy<br>Asthma	2	D

FCGR2B	Fc Fragment Of IgE Receptor IIb	Atopy	NA	D
HLX	H2.0 Like Homeobox	Asthma	2	D
HNMT	Histamine N-Methyltransferase	Dermatitis<br>Asthma	NA	D
IL6R	Interleukin 6 Receptor	Dermatitis	NA	D
IL18	Interleukin 18	Asthma	1	D
INPP4A	Inositol Polyphosphate-4-Phosphatase Type I A	Asthma	1	D
IRAK3	Interleukin 1 Receptor Associated Kinase 3	Asthma	2	D
IRF2	Interferon Regulatory Factor 2	Dermatitis	1	D
MMP9	Matrix Metallopeptidase 9	Asthma<br>Allergy	2	D
MMP12	Matrix Metallopeptidase 12	Asthma	NA	D
NLRP3	NLR Family Pyrin Domain Containing 3	Food allergy	2	D
NOD1	Nucleotide Binding Oligomerization Domain Containing 1	IgE<br>Asthma	1	D
NOD2	Nucleotide Binding Oligomerization Domain Containing 2	Atopy<br>Dermatitis<br>Rhinitis	NA	D
ORAI1†	Calcium Release-Activated Calcium Modulator 1	Dermatitis	1	D
PLA2G4A	Phospholipase A2 Group IVA	Asthma	2	D
PLA2G7	Phospholipase A2 Group VII	Atopy<br>Asthma	2	D
PTGER2	Prostaglandin E Receptor 2	Asthma	1	D
STAT6	Signal Transducer And Activator Of Transcription 6	IgE<br>Dermatitis<br>Asthma	3	D
TLR2	Toll Like Receptor 2	Dermatitis<br>Asthma	1	D
TLR6	Toll Like Receptor 6	Asthma	1	D
TLR9	Toll Like Receptor 9	Dermatitis<br>Asthma	1	D
CCL2	C-C Motif Chemokine Ligand 2	Asthma	NA	E
CCL7	C-C Motif Chemokine Ligand 7	Asthma	1	E
CTLA4†	Cytotoxic T-Lymphocyte Associated Protein 4	Asthma	1	F
EPHX1	Epoxide Hydrolase 1	Asthma	NA	F
ICOS†	Inducible T Cell Costimulator	Allergy	1	F
IL2	Interleukin 2	Allergy	2	F
IL7R†	Interleukin 7 Receptor	Dermatitis	NA	F
IL12RB1†	Interleukin 12 Receptor Subunit Beta 1	Dermatitis	1	F
IL17RB	Interleukin 17 Receptor B	Asthma	1	F
IL21†	Interleukin 21	Asthma	1	F
IL21R†	Interleukin 21 Receptor	IgE	1	F
ITK†	Interleukin 2 Inducible T Cell Kinase	Asthma	1	F
LTA	Lymphotoxin Alpha	Asthma	1	F
PDCD4	Programmed Cell Death 4	Asthma	1	F
PECAM1	Platelet And Endothelial Cell Adhesion	Asthma	NA	F

	Molecule 1			
PPARGC1B	Peroxisome Proliferator-Activated Receptor Gamma Coactivator 1 Beta	Asthma	2	F
S1PR1	Sphingosine-1-Phosphate Receptor 1	Asthma	2	F
TBXA2R	Thromboxane A2 Receptor	IgE<br>Dermatitis<br>Asthma	2	F
TRAF3IP2†	TNF Receptor-Associated Factor 3 Interacting Protein 2	Dermatitis	2	F
ZPBP2	Zona Pellucida Binding Protein 2	Asthma	2	F
CAT	Catalase	Asthma	1	G
SMPD1	Sphingomyelin Phosphodiesterase 1	Allergy	1	G
CD53	Cluster of Differentiation 53 Molecule	Asthma	1	H
DOCK8†	Dedicator Of Cytokinesis 8	IgE	45	H
STK10	Serine/Threonine Kinase 10	Asthma	1	H
TGFB1	Transforming Growth Factor Beta 1	Asthma	NA	H
TYK2†	Tyrosine Kinase 2	IgE	1	H
GSTP1	Glutathione S-Transferase Pi 1	Dermatitis<br>Asthma	NA	I
PGM3†	Phosphoglucomutase 3	Atopy<br>IgE	6	I
CCL5	C-C Motif Chemokine Ligand 5	Dermatitis<br>Asthma	1	J
CYSLTR2	Cysteinyl Leukotriene Receptor 2	Atopy	1	J
IFNG	Interferon Gamma	Atopy	NA	J
IL12RB2	Interleukin 12 Receptor Subunit Beta 2	Atopy	3	J
TBX21	T-Box 21	Asthma	1	J
BDNF	Brain Derived Neurotrophic Factor	Asthma	NA	K
CCL26	C-C Motif Chemokine Ligand 26	Asthma	1	K
CDHR3	Cadherin Related Family Member 3	Asthma	1	K
CFTR†	Cystic Fibrosis Transmembrane Conductance Regulator	Asthma	6	K
CHIA	Chitinase, Acidic	IgE<br>Asthma	6	K
CHIT1	Chitinase 1	Asthma	NA	K
DBH	Dopamine Beta-Hydroxylase	Asthma	NA	K
FLG2	Filaggrin Family Member 2	Dermatitis	1	K
GC	GC, Vitamin D Binding Protein	Asthma	NA	K
GSDMA	Gasdermin A	Asthma	1	K
GSTO2	Glutathione S-Transferase Omega 2	Asthma	NA	K
IL9	Interleukin 9	Dermatitis	1	K
IL31	Interleukin 31	Dermatitis	1	K
KLK7	Kallikrein Related Peptidase 7	Dermatitis	1	K
NOS1	Nitric Oxide Synthase 1	Asthma	1	K
NOS2	Nitric Oxide Synthase 2	Atopy	NA	K
PTGDR	Prostaglandin D2 Receptor	Asthma<br>Allergy	4	K
PTGDR2	Prostaglandin D2 Receptor 2	Asthma	2	K
SCGB1A1	Secretoglobin Family 1A Member 1	Asthma	1	K

SPINK5†	Serine Peptidase Inhibitor, Kazal Type 5	Atopy<br>Asthma	3	K
TCHHL1	Trichohyalin Like 1	Dermatitis	1	K
TMEM79	Transmembrane Protein 79	Dermatitis	1	K
TRPV1	Transient Receptor Potential Cation Channel Subfamily V Member 1	Asthma	1	K
ADAM33	ADAM Metallopeptidase Domain 33	Asthma	NA	bin
ATG5	Autophagy Related 5	Asthma	2	bin
CASP8†	Caspase 8	Asthma	NA	bin
CD38	Cluster of Differentiation 38 Molecule	Asthma	1	bin
CEP68	Centrosomal Protein 68	Asthma	1	bin
CSF2	Colony Stimulating Factor 2	Dermatitis	1	bin
CSTA	Cystatin A	Dermatitis	1	bin
DEFB1	Defensin Beta 1	Dermatitis	NA	bin
F2RL1	F2R Like Trypsin Receptor 1	Atopy	1	bin
GRASP	General Receptor For Phosphoinositides 1 Associated Scaffold Protein	Asthma	1	bin
GSTM1	Glutathione S-Transferase Mu 1	Dermatitis<br>Asthma	NA	bin
HAVCR1	Hepatitis A Virus Cellular Receptor 1	Asthma	3	bin
HMGB1	High Mobility Group Box 1	Dermatitis	3	bin
IL4R	Interleukin 4 Receptor	Atopy<br>Dermatitis<br>Asthma	6	bin
IL10†	Interleukin 10	Asthma	NA	bin
IL12B†	Interleukin 12B	Dermatitis<br>Asthma	2	bin
IL17F†	Interleukin 17F	Asthma	1	bin
KAT6A	Lysine Acetyltransferase 6A	Food allergy	1	bin
LPL	Lipoprotein Lipase	Dermatitis	NA	bin
LTC4S	Leukotriene C4 Synthase	Asthma<br>Allergy	2	bin
MAP3K1	Mitogen-Activated Protein Kinase 1	Asthma	1	bin
NAT2	N-Acetyltransferase 2	Asthma	2	bin
NFKBIA	Nuclear Factor Kappa B Inhibitor Alpha	Asthma	1	bin
NR3C1	Nuclear Receptor Subfamily 3 Group C Member 1	Asthma	NA	bin
ORMDL3	Orosomucoid Like 3	Asthma	3	bin
PAG1	Phosphoprotein Membrane Anchor With Glycosphingolipid Microdomains 1	Allergy	1	bin
PHF11	PHD Finger Protein 11	Dermatitis<br>Asthma	2	bin
PTGS2	Prostaglandin-Endoperoxide Synthase 2	Atopy<br>Asthma	NA	bin
RBFOX1	RNA Binding Fox-1 Homolog 1	Food allergy	2	bin
SART1	U4/U6.U5 Tri-SnRNP-Associated Protein 1	Atopy	1	bin
SCGB3A2	Secretoglobin Family 3A Member 2	Asthma	1	bin
STAT3†	Signal Transducer And Activator Of Transcription 3	IgE	107	bin

TLR1	Toll Like Receptor 1	Asthma	NA	bin
TNF	Tumor Necrosis Factor	Asthma	NA	bin
TNFRSF13B[†]	Tumor Necrosis Factor Receptor Superfamily Member 13B	Asthma	NA	bin
TSLP	Thymic Stromal Lymphopoietin	Asthma	1	bin
ACE	Angiotensin I Converting Enzyme	Asthma	NA	NA
CCL3L1	C-C Motif Chemokine Ligand 3 Like 1	Asthma	NA	NA
CHI3L1	Chitinase 3 Like 1	Asthma	1	NA
CYP2C19	Cytochrome P450 Family 2 Subfamily C Member 19	Asthma	NA	NA
CYP2J2	Cytochrome P450 Family 2 Subfamily J Member 2	Asthma	NA	NA
ERBIN	Erb-B2 Interacting Protein	IgE	1	NA
FCGR2A	Fc Fragment Of IgE Receptor IIa	Atopy<br>Allergy	1	NA
FLG	Filaggrin	Dermatitis<br>Food allergy<br>Asthma	62	NA
FLG10.2	Fillagrin Alternative Isoform, Repeat 10.2	Dermatitis	NA	NA
FLG11	Fillagrin Alternative Isoform, Repeat 11	Dermatitis<br>Asthma	1	NA
GSDMB	Gasdermin B	Asthma	4	NA
LCE3C	Late Cornified Envelope 3C	Dermatitis	NA	NA
MUC7	Mucin 7, Secreted	Asthma	1	NA
TLR10	Toll Like Receptor 10	Asthma<br>Rhinitis	1	NA
VSTM1	V-Set And Transmembrane Domain Containing 1	Dermatitis	1	NA

Abbreviations: NA, not available

* Human atopy-related genes identified in the Human Gene Mutation Database (HGMD) were not available for clustering because they could not be identified in the mouse immune system as used in ImmGen.

[†] Overlapping genes between atopy-related gene list and PID-related gene list (n=22).
