# Supplementary material for: Molecular clustering of genes related to the atopic syndrome: Towards a more tailored approach and personalized medicine?
Source: Clin Transl Allergy. 2019 Jul 10;9:34. doi: 10.1186/s13601-019-0273-8 (PMC6617681; doi:10.1186/s13601-019-0273-8)
Supplement: Supplementary file 2 — Additional file 2: Table S2. (a) Ingenuity Pathway Analysis – top three pathways of all atopy-related genes (n = 160); (b) Ingenuity Pathway Analysis - all atopy-related genes (n = 160); (c) Ingenuity Pathway Analysis – top 3 pathways of atopy-related genes without ‘bin’ genes (n = 108); (d) Ingenuity Pathway Analysis – atopy-related genes without ‘bin’ genes (n = 108). [file 13601_2019_273_MOESM2_ESM.pdf]

Additional File 2: Table S2a. Ingenuity pathway analysis – top three pathways of all atopy genes (n=160)

Pathways	-log (p-value)[1]	Ratio[2]	Molecules
T Helper Cell Differentiation	2,95E01	3,01E-01	*IL21, STAT6, IFNG, IL4R, IL12RB1, IL10, IL21R, IL6R, IL12RB2, STAT3, TBX21, IL13, IL18, IL2, TGFB1, IL12B, NGFR, ICOS, CD86, IL17F, TNF, IL4*
Th1 and Th2 Activation Pathway	2,84E01	1,51E-01	*IL1RL1, IL12RB1, IL31, TBX21, IL2, TGFB1, IL9, IL4, STAT6, IFNG, PTGDR2, IL4R, IL10, HAVCR1, IL6R, TYK2, IL12RB2, STAT3, TLR9, IL13, TSLP, IL18, IL17RB, IL12B, LTA, ICOS, S1PR1, CD86*
Th2 Pathway	2,49E01	1,6E-01	*STAT6, IFNG, IL4R, PTGDR2, IL12RB1, IL10, HAVCR1, IL1RL1, TYK2, IL12RB2, IL31, TLR9, IL13, TBX21, TSLP, IL17RB, IL2, TGFB1, IL12B, ICOS, IL9, S1PR1, CD86, IL4*

[1]The –log (p-value) was calculated using a Fisher's exact test to determine the probability that the association between the atopy-related genes and the pathways is explained by a random chance alone. A –log (p-value) equal to or greater than 1.3, corresponding to a p-value of 0.05, was considered statistically significant.
[2]The ratio is calculated by the atopy-related genes found in each pathway divided by the total number of molecules in that pathway.

Additional File 2: Table S2b. Ingenuity pathway analysis - all atopy genes (n=160)

Pathways	Molecules
T Helper Cell Differentiation	*IL21, STAT6, IFNG, IL4R, IL12RB1, IL10, IL21R, IL6R, IL12RB2, STAT3, TBX21, IL13, IL18, IL2, TGFB1, IL12B, NGFR, ICOS, CD86, IL17F, TNF, IL4*
Th1 and Th2 Activation Pathway	*IL1RL1, IL12RB1, IL31, TBX21, IL2, TGFB1, IL9, IL4, STAT6, IFNG, PTGDR2, IL4R, IL10, HAVCR1, IL6R, TYK2, IL12RB2, STAT3, TLR9, IL13, TSLP, IL18, IL17RB, IL12B, LTA, ICOS, S1PR1, CD86*
Th2 Pathway	*STAT6, IFNG, IL4R, PTGDR2, IL12RB1, IL10, HAVCR1, IL1RL1, TYK2, IL12RB2, IL31, TLR9, IL13, TBX21, TSLP, IL17RB, IL2, TGFB1, IL12B, ICOS, IL9, S1PR1, CD86, IL4*
TREM1 Signaling	*NLRP3, IL10, IL1RL1, STAT3, FCGR2B, TLR9, TLR2, TLR10, IL18, NOD2, CCL2, TLR1, TLR6, NOD1, CD86, CSF2, TNF, CCL7*
Neuroinflammation Signaling Pathway	*BDNF, CCL5, HMGB1, TLR10, CCL2, TGFB1, TLR1, NOS2, CASP8, IL4, IFNG, NLRP3, IL10, IL6R, TYK2, IRAK3, TLR9, CSF1R, TLR2, PLA2G4A, IL18, IL12B, TLR6, CD86, PTGS2, TNF, SLC6A12, MMP9*
Altered T Cell and B Cell Signaling in Rheumatoid Arthritis	*IL21, IFNG, IL10, TLR9, TLR2, TLR10, IL18, IL12B, TGFB1, IL2, LTA, TLR1, TLR6, CD86, TNFRSF13B, CSF2, TNF, IL4*
Role of Pattern Recognition Receptors in Recognition of Bacteria and Viruses	*IFNG, NLRP3, IL10, CCL5, IL13, TLR9, TLR2, IL18, NOD2, IL12B, TGFB1, IL2, LTA, TLR1, TLR6, NOD1, CSF2, IL17F, TNF, IL4*
Differential Regulation of Cytokine Production in Intestinal Epithelial Cells by IL-17A and IL-17F	*IFNG, CCL2, IL10, IL12B, IL9, DEFB1, CCL5, CSF2, IL13, IL17F, TNF*
Communication between Innate and Adaptive Immune Cells	*IFNG, IL10, CCL5, TLR9, TLR2, TLR10, IL18, IL2, IL12B, TLR6, TLR1, CD86, TNFRSF13B, CSF2, TNF, IL4*
Th17 Activation Pathway	*IL21, IFNG, IL12RB1, IL10, IL21R, IL6R, TYK2, DEFB1, IL12RB2, IRAK3, STAT3, IL12B, PTGER2, CSF2, IL17F*
HMGB1 Signaling	*IFNG, IL13, TLR9, HMGB1, IL18, CCL2, IL12B, TGFB1, IL2, NGFR, KAT6A, LTA, CSF2, SERPINE1, IL17F, TNF, IL4*
Role of Macrophages, Fibroblasts and Endothelial Cells in Rheumatoid Arthritis	*IL10, IL1RL1, IL6R, STAT3, IRAK3, CCL5, TLR9, TLR2, TLR10, IL18, NFKBIA, TRAF3IP2, F2RL1, CCL2, TGFB1, LTA, NGFR, TLR1, TLR6, CSF2, NOS2, TNF*
Differential Regulation of Cytokine Production in Macrophages and T Helper Cells by IL-17A and IL-17F	*CCL2, IL10, IL12B, IL9, CCL5, CSF2, IL13, IL17F, TNF*
Th1 Pathway	*IFNG, IL12RB1, IL10, IL6R, TYK2, IL12RB2, STAT3, TBX21, TLR9, IL18, IL12B, IL2, LTA, ICOS, CD86, IL4*
Role of Cytokines in Mediating Communication between Immune Cells	*IL21, IFNG, IL18, IL2, TGFB1, IL10, IL12B, CSF2, IL13, IL17F, TNF, IL4*
Glucocorticoid Receptor Signaling	*IFNG, IL10, MAP3K1, STAT3, CCL5, CCL11, IL13, TLR9, NR3C1, HMGB1, SCGB1A1, NFKBIA, CCL2, IL2, TGFB1, PTGS2, CSF2, NOS2, SERPINE1, TNF, ADRB2, IL4*
Toll-like Receptor Signaling	*TLR2, IL18, TLR10, NFKBIA, IL1RL1, IL12B, TLR1, MAP3K1, TLR6, CD14, IRAK3, TLR9, TNF*

Hepatic Cholestasis	*IFNG, IL1RL1, IRAK3, IL13, IL18, NFKBIA, IL12B, IL2, TGFB1, NGFR, LTA, CD14, CSF2, IL17F, TNF, IL4*
IL-10 Signaling	*IL18, IL4R, NFKBIA, IL1RL1, IL10, FCGR2A, TYK2, CD14, STAT3, FCGR2B, TNF*
IL-12 Signaling and Production in Macrophages	*TLR2, STAT6, IFNG, IL18, IL12B, TGFB1, IL10, IL12RB1, SERPINA1, IL12RB2, NOS2, TLR9, TNF, IL4*
Granulocyte Adhesion and Diapedesis	*IL18, SELP, CCL3L1, CCL2, IL1RL1, NGFR, PECAM1, CCL26, CCL5, MMP12, HRH4, CCL11, TNF, MMP9, CCL7*
LXR/RXR Activation	*IL18, CCL2, IL1RL1, NGFR, LPL, CD14, SERPINA1, PTGS2, GC, NOS2, TNF, MMP9, CCL7*
Hepatic Fibrosis / Hepatic Stellate Cell Activation	*IFNG, IL4R, CCL2, TGFB1, IL1RL1, IL10, NGFR, IL6R, CD14, COL6A5, CCL5, SERPINE1, TNF, MMP9, IL4*
Atherosclerosis Signaling	*PLA2G4A, IFNG, IL18, CCL2, SELP, TGFB1, LPL, SERPINA1, ALOX5, CCL11, TNF, PLA2G7, MMP9*
Role of JAK1 and JAK3 in γc Cytokine Signaling	*IL7R, IL21, STAT6, IL4R, IL2, IL21R, IL9, STAT3, TLR9, TSLP, IL4*
Eicosanoid Signaling	*PLA2G4A, PTGDR, LTC4S, TBXA2R, CYSLTR1, PTGS2, PTGER2, ALOX5, CYSLTR2, PLA2G7*
Colorectal Cancer Metastasis Signaling	*IFNG, IL6R, TYK2, STAT3, TLR9, TLR2, TLR10, TGFB1, TLR6, TLR1, PTGS2, PTGER2, NOS2, MMP12, TNF, MMP9*
Crosstalk between Dendritic Cells and Natural Killer Cells	*IFNG, IL18, IL2, IL12B, LTA, CD86, CSF2, TLR9, TNF, IL4*
NF-κB Signaling	*TLR2, TLR10, IL18, NFKBIA, NGFR, LTA, TLR6, TLR1, MAP3K1, IRAK3, CASP8, TLR9, TNF*
Dendritic Cell Maturation	*TLR2, IL18, NFKBIA, FCGR2A, IL10, IL12B, LTA, NGFR, CD86, CSF2, TLR9, FCGR2B, TNF*
Type I Diabetes Mellitus Signaling	*IFNG, NFKBIA, IL2, IL12B, NGFR, LTA, CD86, NOS2, CASP8, TNF*
Agranulocyte Adhesion and Diapedesis	*IL18, SELP, CCL2, CCL3L1, PECAM1, CCL26, CCL5, MMP12, CCL11, TNF, MMP9, CCL7*
Production of Nitric Oxide and Reactive Oxygen Species in Macrophages	*TLR2, IFNG, NFKBIA, NGFR, CAT, MAP3K1, TYK2, SERPINA1, NOS2, TLR9, TNF, IL4*
Role of Hypercytokinemia/hyperchemokinemia in the Pathogenesis of Influenza	*IFNG, IL18, CCL2, IL12B, IL9, CCL5, TNF*
MSP-RON Signaling Pathway	*TLR2, IFNG, KLK7, CCL2, IL12B, NOS2, TLR9, TNF*
LPS/IL-1 Mediated Inhibition of RXR Function	*GSTM1, IL18, IL1RL1, NGFR, CAT, MAP3K1, PPARGC1B, CD14, GSTO2, TNF, GSTP1, CYP2C19*
Role of Osteoblasts, Osteoclasts and Chondrocytes in Rheumatoid Arthritis	*IFNG, IL18, NFKBIA, IL1RL1, TGFB1, IL10, NGFR, CSF2, TLR9, TNF, CSF1R, IL4*
Fc Epsilon RI Signaling	*BTK, PLA2G4A, FCER1A, MS4A2, CSF2, TLR9, IL13, TNF, IL4*
IL-6 Signaling	*IL18, NFKBIA, IL1RL1, NGFR, IL6R, CD14, STAT3, TLR9, TNF*
Acute Phase Response Signaling	*IL18, NFKBIA, NGFR, MAP3K1, IL6R, SERPINA1, STAT3, SERPINE1, TNF, NR3C1*
Role of IL-17A in Arthritis	*NFKBIA, CCL2, PTGS2, CCL5, TLR9, NOS2, CCL7*
iNOS Signaling	*IFNG, NFKBIA, TYK2, CD14, IRAK3, NOS2*
Role of NFAT in Regulation of the Immune Response	*BTK, NFKBIA, FCGR2A, FCER1A, MS4A2, CD86, FCGR2B, TLR9, ITK, ORAI1*
Hematopoiesis from Pluripotent Stem Cells	*IL2, IL10, IL12B, IL9, CSF2, IL4*

Airway Inflammation in Asthma	*IL13, TNF, IL4*
Role of MAPK Signaling in the Pathogenesis of Influenza	*IFNG, PLA2G4A, CCL2, PTGS2, CCL5, PLA2G7, TNF*
IL-15 Signaling	*STAT6, TYK2, STAT3, TLR9, CSF2, TNF, IL4*
Osteoarthritis Pathway	*HMGB1, TLR2, IL1RL1, TGFB1, PTGS2, NOS2, MMP12, CASP8, TNF, MMP9*
Tec Kinase Signaling	*BTK, STAT6, TYK2, FCER1A, MS4A2, STAT3, TLR9, TNF, ITK*
Renin-Angiotensin Signaling	*CCL2, MAP3K1, CCL5, STAT3, PTGER2, TLR9, TNF, ACE*
IL-17 Signaling	*TRAF3IP2, CCL2, PTGS2, CCL11, TLR9, NOS2, IL17F*
MIF-mediated Glucocorticoid Regulation	*PLA2G4A, NFKBIA, CD14, PTGS2, NR3C1*
CD28 Signaling in T Helper Cells	*NFKBIA, ARPC1B, IL2, MAP3K1, CD86, TLR9, CTLA4, ITK*
Phagosome Formation	*TLR2, TLR10, FCGR2A, TLR6, TLR1, FCER1A, TLR9, FCGR2B*
Systemic Lupus Erythematosus Signaling	*IL18, IL2, IL10, FCGR2A, IL6R, CD86, FCGR2B, TLR9, SART1, TNF*
STAT3 Pathway	*IL4R, IL17RB, TGFB1, NGFR, TYK2, IL9, STAT3*
Inflammasome pathway	*IL18, NOD2, NLRP3, CASP8*
MIF Regulation of Innate Immunity	*PLA2G4A, NFKBIA, CD14, PTGS2, NOS2*
Role of IL-17F in Allergic Inflammatory Airway Diseases	*TRAF3IP2, CCL2, CSF2, IL17F, CCL7*
T Cell Receptor Signaling	*BTK, NFKBIA, PAG1, MAP3K1, TLR9, CTLA4, ITK*
VDR/RXR Activation	*IFNG, IL2, IL1RL1, CD14, CCL5, CSF2*
IL-17A Signaling in Airway Cells	*TRAF3IP2, NFKBIA, TYK2, STAT3, TLR9, CCL11*
Graft-versus-Host Disease Signaling	*IFNG, IL18, IL2, CD86, TNF*
Allograft Rejection Signaling	*IFNG, IL2, IL10, CD86, TNF, IL4*
TNFR2 Signaling	*NFKBIA, LTA, MAP3K1, TNF*
IL-4 Signaling	*STAT6, IL4R, TYK2, TLR9, NR3C1, IL4*
Hematopoiesis from Multipotent Stem Cells	*IL2, CSF2, IL4*
Glutathione-mediated Detoxification	*GSTM1, LTC4S, GSTO2, GSTP1*
Ceramide Signaling	*NGFR, MAP3K1, S1PR1, SMPD1, TLR9, TNF*
Endothelin-1 Signaling	*NOS1, PLA2G4A, PTGS2, PTGER2, TLR9, NOS2, CASP8, PLA2G7*
PPAR Signaling	*IL18, NFKBIA, IL1RL1, NGFR, PTGS2, TNF*
IL-17A Signaling in Fibroblasts	*TRAF3IP2, NFKBIA, CCL2, CCL7*
Antioxidant Action of Vitamin C	*PLA2G4A, NFKBIA, GSTO2, CSF2, TNF, PLA2G7*
Endocannabinoid Cancer Inhibition Pathway	*NOS1, TWIST1, SMPD1, TRPV1, CASP8, TLR9, NOS2*
Role of PKR in Interferon Induction and Antiviral Response	*IFNG, NFKBIA, CASP8, TNF*
p38 MAPK Signaling	*PLA2G4A, IL18, IL1RL1, TGFB1, IRAK3, TNF*
Pancreatic Adenocarcinoma Signaling	*TGFB1, TYK2, STAT3, PTGS2, TLR9, MMP9*
CD40 Signaling	*NFKBIA, LTA, STAT3, PTGS2, TLR9*
IL-9 Signaling	*IL9, STAT3, TLR9, TNF*
Xenobiotic Metabolism Signaling	*GSTM1, CAT, MAP3K1, GSTO2, NOS2, TLR9, TNF, GSTP1, CYP2C19*
Autoimmune Thyroid Disease Signaling	*IL10, IL2, CD86, IL4*

Primary Immunodeficiency Signaling	*IL7R, BTK, ICOS, TNFRSF13B*
TNFR1 Signaling	*NFKBIA, MAP3K1, CASP8, TNF*
Citrulline-Nitric Oxide Cycle	*NOS1, NOS2*
B Cell Receptor Signaling	*BTK, NFKBIA, FCGR2A, PAG1, MAP3K1, FCGR2B, TLR9*
Gα12/13 Signaling	*BTK, NFKBIA, F2RL1, TBXA2R, MAP3K1, TLR9*
NRF2-mediated Oxidative Stress Response	*GSTM1, CAT, MAP3K1, GSTO2, TLR9, GSTP1, EPHX1*
Role of JAK1, JAK2 and TYK2 in Interferon Signaling	*IFNG, TYK2, STAT3*
Role of JAK family kinases in IL-6-type Cytokine Signaling	*IL6R, TYK2, STAT3*
Leukocyte Extravasation Signaling	*BTK, CTNNA3, PECAM1, MMP12, TLR9, MMP9, ITK*
Type II Diabetes Mellitus Signaling	*NFKBIA, NGFR, MAP3K1, SMPD1, TLR9, TNF*
SPINK1 Pancreatic Cancer Pathway	*KLK7, F2RL1, TGFB1, MMP12*
Induction of Apoptosis by HIV1	*NFKBIA, NGFR, CASP8, TNF*
G-Protein Coupled Receptor Signaling	*NFKBIA, PTGDR, TBXA2R, S1PR1, STAT3, PTGER2, TLR9, ADRB2*
Activation of IRF by Cytosolic Pattern Recognition Receptors	*NFKBIA, IL10, LTA, TNF*
PXR/RXR Activation	*GSTM1, TNF, NR3C1, CYP2C19*
Airway Pathology in Chronic Obstructive Pulmonary Disease	*TNF, MMP9*
iCOS-iCOSL Signaling in T Helper Cells	*NFKBIA, IL2, ICOS, TLR9, ITK*
HIF1α Signaling	*NOS1, NOS2, MMP12, TLR9, MMP9*
FXR/RXR Activation	*IL18, LPL, SERPINA1, GC, TNF*
Chemokine Signaling	*CCL2, CCL5, CCL11, CCL7*
Role of PI3K/AKT Signaling in the Pathogenesis of Influenza	*IFNG, NFKBIA, CCL5, TLR9*
SPINK1 General Cancer Pathway	*IL6R, TYK2, STAT3, TLR9*
April Mediated Signaling	*NFKBIA, MAP3K1, TNFRSF13B*
Adipogenesis pathway	*ATG5, TGFB1, KAT6A, LPL, TNF*
Regulation of the Epithelial-Mesenchymal Transition Pathway	*TGFB1, TYK2, TWIST1, STAT3, TLR9, MMP9*
Oncostatin M Signaling	*TYK2, STAT3, CHI3L1*
CCR3 Signaling in Eosinophils	*PLA2G4A, MYLK, CCL26, TLR9, CCL11*
PI3K Signaling in B Lymphocytes	*BTK, IL4R, NFKBIA, FCGR2B, IL4*
B Cell Activating Factor Signaling	*NFKBIA, MAP3K1, TNFRSF13B*
p70S6K Signaling	*BTK, IL4R, F2RL1, TLR9, IL4*
Small Cell Lung Cancer Signaling	*NOS1, NFKBIA, PTGS2, TLR9*
Regulation of IL-2 Expression in Activated and Anergic T Lymphocytes	*NFKBIA, TGFB1, IL2, MAP3K1*
Aryl Hydrocarbon Receptor Signaling	*GSTM1, TGFB1, GSTO2, TNF, GSTP1*
Leukotriene Biosynthesis	*LTC4S, ALOX5*

JAK/Stat Signaling	*STAT6, TYK2, STAT3, TLR9*
PEDF Signaling	*NFKBIA, BDNF, TLR9, CASP8*
Death Receptor Signaling	*NFKBIA, CASP8, TNF, PARP1*
Superpathway of Citrulline Metabolism	*NOS1, NOS2*
Apoptosis Signaling	*NFKBIA, CASP8, TNF, PARP1*
PDGF Signaling	*MAP3K1, TYK2, STAT3, TLR9*
Granzyme B Signaling	*CASP8, PARP1*
cAMP-mediated signaling	*PTGDR, TBXA2R, S1PR1, STAT3, PTGER2, ADRB2*
CD27 Signaling in Lymphocytes	*NFKBIA, MAP3K1, CASP8*
PKCθ Signaling in T Lymphocytes	*NFKBIA, IL2, MAP3K1, CD86, TLR9*
Apelin Cardiomyocyte Signaling Pathway	*TGFB1, CAT, MYLK, TLR9*
Retinoic acid Mediated Apoptosis Signaling	*IFNG, CASP8, PARP1*
Apelin Cardiac Fibroblast Signaling Pathway	*TGFB1, SERPINE1*
HGF Signaling	*MAP3K1, STAT3, PTGS2, TLR9*
Neuroprotective Role of THOP1 in Alzheimer's Disease	*IFNG, KLK7, MMP9, ACE*
PPARα/RXRα Activation	*NFKBIA, IL1RL1, TGFB1, LPL, CYP2C19*
Lymphotoxin β Receptor Signaling	*NFKBIA, LTA, TLR9*
NGF Signaling	*NGFR, MAP3K1, SMPD1, TLR9*
IL-22 Signaling	*TYK2, STAT3*
Glutathione Redox Reactions I	*GSTM1, GSTP1*
Sphingosine-1-phosphate Signaling	*S1PR1, SMPD1, CASP8, TLR9*
EGF Signaling	*MAP3K1, STAT3, TLR9*
CNTF Signaling	*TYK2, STAT3, TLR9*
IL-17A Signaling in Gastric Cells	*CCL5, TNF*
Bupropion Degradation	*CYP2J2, CYP2C19*
ILK Signaling	*PTGS2, NOS2, TLR9, TNF, MMP9*
Antiproliferative Role of TOB in T Cell Signaling	*IL2, TGFB1*
Adrenomedullin signaling pathway	*IL18, LTA, MYLK, TLR9, TNF*
IL-8 Signaling	*IL9, PTGS2, IRAK3, TLR9, MMP9*
ErbB2-ErbB3 Signaling	*TYK2, STAT3, TLR9*
Corticotropin Releasing Hormone Signaling	*NOS1, BDNF, PTGS2, NOS2*
GM-CSF Signaling	*STAT3, CSF2, TLR9*
Acetone Degradation I (to Methylglyoxal)	*CYP2J2, CYP2C19*
Human Embryonic Stem Cell Pluripotency	*BDNF, TGFB1, S1PR1, TLR9*
Apelin Adipocyte Signaling Pathway	*GSTM1, CAT, GSTP1*
Neurotrophin/TRK Signaling	*BDNF, NGFR, TLR9*
FcγRIIB Signaling in B Lymphocytes	*BTK, FCGR2B, TLR9*

Inhibition of Angiogenesis by TSP1	*TGFB1, MMP9*
Role of JAK2 in Hormone-like Cytokine Signaling	*TYK2, STAT3*
Macropinocytosis Signaling	*CD14, TLR9, CSF1R*
Coagulation System	*SERPINA1, SERPINE1*
TWEAK Signaling	*NFKBIA, CASP8*
IL-3 Signaling	*STAT6, STAT3, TLR9*
Prolactin Signaling	*STAT3, TLR9, NR3C1*
B Cell Development	*IL7R, CD86*
Interferon Signaling	*IFNG, TYK2*
Relaxin Signaling	*NFKBIA, NOS2, TLR9, MMP9*
FGF Signaling	*MAP3K1, STAT3, TLR9*
IL-7 Signaling Pathway	*IL7R, IFNG, TLR9*
Fcγ Receptor-mediated Phagocytosis in Macrophages and Monocytes	*ARPC1B, FCGR2A, CSF2*
IL-1 Signaling	*NFKBIA, MAP3K1, IRAK3*
LPS-stimulated MAPK Signaling	*NFKBIA, CD14, TLR9*
NF-κB Activation by Viruses	*NFKBIA, MAP3K1, TLR9*
FLT3 Signaling in Hematopoietic Progenitor Cells	*STAT6, STAT3, TLR9*
Cardiac Hypertrophy Signaling	*TGFB1, MAP3K1, IL6R, TLR9, ADRB2*
Inhibition of Matrix Metalloproteases	*MMP12, MMP9*
Phospholipase C Signaling	*BTK, PLA2G4A, FCGR2A, FCGR2B, ITK*
Tight Junction Signaling	*TGFB1, NGFR, MYLK, TNF*
Acute Myeloid Leukemia Signaling	*STAT3, TLR9, CSF1R*
CTLA4 Signaling in Cytotoxic T Lymphocytes	*CD86, TLR9, CTLA4*
Estrogen Biosynthesis	*CYP2J2, CYP2C19*
RANK Signaling in Osteoclasts	*NFKBIA, MAP3K1, TLR9*
Prostate Cancer Signaling	*NFKBIA, TLR9, GSTP1*
Role of RIG1-like Receptors in Antiviral Innate Immunity	*NFKBIA, CASP8*
Germ Cell-Sertoli Cell Junction Signaling	*TGFB1, MAP3K1, TLR9, TNF*
Synaptic Long Term Depression	*NOS1, PLA2G4A, NOS2, PLA2G7*
Cholecystokinin/Gastrin-mediated Signaling	*IL18, PTGS2, TNF*
PAK Signaling	*MYLK, TLR9, TNF*
Sertoli Cell-Sertoli Cell Junction Signaling	*NOS1, MAP3K1, NOS2, TNF*
Gαs Signaling	*PTGDR, PTGER2, ADRB2*
Amyotrophic Lateral Sclerosis Signaling	*NOS1, CAT, TLR9*
UVA-Induced MAPK Signaling	*SMPD1, TLR9, PARP1*
Mouse Embryonic Stem Cell Pluripotency	*TYK2, STAT3, TLR9*
Transcriptional Regulatory Network in Embryonic Stem	*KAT6A, STAT3*

Cells	
Rac Signaling	*ARPC1B, MAP3K1, TLR9*
Gαi Signaling	*TBXA2R, S1PR1, STAT3*
Sirtuin Signaling Pathway	*ATG5, STAT3, NOS2, TNF, PARP1*
Nicotine Degradation III	*CYP2J2, CYP2C19*
Role of NANOG in Mammalian Embryonic Stem Cell Pluripotency	*TYK2, STAT3, TLR9*
fMLP Signaling in Neutrophils	*NFKBIA, ARPC1B, TLR9*
Role of Tissue Factor in Cancer	*F2RL1, CSF2, TLR9*
PI3K/AKT Signaling	*NFKBIA, TYK2, PTGS2*
Ephrin A Signaling	*NGFR, TLR9*
Protein Kinase A Signaling	*NFKBIA, TGFB1, NGFR, MAP3K1, MYLK, PTGS2*
GP6 Signaling Pathway	*BTK, COL6A5, TLR9*
Phospholipases	*PLA2G4A, PLA2G7*
autophagy	*ATG5, ACE*
Iron homeostasis signaling pathway	*IL6R, TYK2, STAT3*
Melatonin Degradation I	*CYP2J2, CYP2C19*
Nicotine Degradation II	*CYP2J2, CYP2C19*
Actin Cytoskeleton Signaling	*ARPC1B, CD14, MYLK, TLR9*
Superpathway of Melatonin Degradation	*CYP2J2, CYP2C19*
IL-2 Signaling	*IL2, TLR9*
Thrombopoietin Signaling	*STAT3, TLR9*
Ovarian Cancer Signaling	*PTGS2, TLR9, MMP9*
Myc Mediated Apoptosis Signaling	*TLR9, CASP8*
Glioma Invasiveness Signaling	*TLR9, MMP9*
Gαq Signaling	*BTK, NFKBIA, TLR9*
Axonal Guidance Signaling	*ARPC1B, BDNF, NGFR, TLR9, MMP9, ACE*
Antiproliferative Role of Somatostatin Receptor 2	*NOS1, TLR9*
Cdc42 Signaling	*ARPC1B, MYLK, ITK*
Angiopoietin Signaling	*NFKBIA, TLR9*
Growth Hormone Signaling	*STAT3, TLR9*
Leptin Signaling in Obesity	*STAT3, TLR9*
Erythropoietin Signaling	*NFKBIA, TLR9*
Renal Cell Carcinoma Signaling	*TGFB1, TLR9*
Regulation of Actin-based Motility by Rho	*ARPC1B, MYLK*
OX40 Signaling Pathway	*NFKBIA, IL2*
TGF-β Signaling	*TGFB1, SERPINE1*
Bladder Cancer Signaling	*MMP12, MMP9*
VEGF Family Ligand-Receptor Interactions	*PLA2G4A, TLR9*
Sumoylation Pathway	*NFKBIA, NR3C1*
Molecular Mechanisms of Cancer	*NFKBIA, TGFB1, TYK2, TLR9, CASP8*
RAR Activation	*TGFB1, MAP3K1, PARP1*
CDK5 Signaling	*BDNF, NGFR*
3-phosphoinositide Biosynthesis	*ICOS, CD86, TLR9*

ERK/MAPK Signaling	*PLA2G4A, STAT3, TLR9*
Clathrin-mediated Endocytosis Signaling	*ARPC1B, SERPINA1, TLR9*
SAPK/JNK Signaling	*MAP3K1, TLR9*
IGF-1 Signaling	*STAT3, TLR9*
Chronic Myeloid Leukemia Signaling	*TGFB1, TLR9*
Neuropathic Pain Signaling In Dorsal Horn Neurons	*BDNF, TLR9*
AMPK Signaling	*CFTR, TLR9, ADRB2*
Telomerase Signaling	*IL2, TLR9*
Integrin Signaling	*ARPC1B, MYLK, TLR9*
Circadian Rhythm Signaling	*ADCYAP1R1*
Role of NFAT in Cardiac Hypertrophy	*TGFB1, MAP3K1, TLR9*
RhoA Signaling	*ARPC1B, MYLK*
Apelin Endothelial Signaling Pathway	*CCL2, TLR9*
Sperm Motility	*PLA2G4A, PLA2G7*
Endocannabinoid Developing Neuron Pathway	*STAT3, TLR9*
Natural Killer Cell Signaling	*FCGR2A, TLR9*
Superpathway of Inositol Phosphate Compounds	*ICOS, CD86, TLR9*
14-3-3-mediated Signaling	*TLR9, TNF*
Huntington's Disease Signaling	*BDNF, TLR9, CASP8*
Signaling by Rho Family GTPases	*ARPC1B, MYLK, TLR9*
Phagosome Maturation	*NOS1, ACE*
Mitochondrial Dysfunction	*CAT, CASP8*
eNOS Signaling	*TLR9, CASP8*
Ephrin Receptor Signaling	*ARPC1B, STAT3*
Thrombin Signaling	*MYLK, TLR9*
Opioid Signaling Pathway	*NOS1, NFKBIA*
GPCR-Mediated Integration of Enteroendocrine Signaling Exemplified by an L Cell	*ADRB2*
Actin Nucleation by ARP-WASP Complex	*ARPC1B*
Remodeling of Epithelial Adherens Junctions	*ARPC1B*
RhoGDI Signaling	*ARPC1B*
Epithelial Adherens Junction Signaling	*ARPC1B*
Thyroid Cancer Signaling	*BDNF*
G Beta Gamma Signaling	*BTK*
Tumoricidal Function of Hepatic Natural Killer Cells	*CASP8*
Cytotoxic T Lymphocyte-mediated Apoptosis of Target Cells	*CASP8*
Superoxide Radicals Degradation	*CAT*

Ethanol Degradation IV	*CAT*
Pathogenesis of Multiple Sclerosis	*CCL5*
CCR5 Signaling in Macrophages	*CCL5*
Nur77 Signaling in T Lymphocytes	*CD86*
Catecholamine Biosynthesis	*DBH*
Neuregulin Signaling	*ERBIN*
HIPPO signaling	*FRMD6*
Ascorbate Recycling (Cytosolic)	*GSTO2*
Vitamin-C Transport	*GSTO2*
Histamine Degradation	*HNMT*
Role of Lipids/Lipid Rafts in the Pathogenesis of Influenza	*IFNG*
Antigen Presentation Pathway	*IFNG*
Role of Wnt/GSK-3β Signaling in the Pathogenesis of Influenza	*IFNG*
Role of BRCA1 in DNA Damage Response	*IFNG*
Protein Ubiquitination Pathway	*IFNG*
3-phosphoinositide Degradation	*INPP4A*
Intrinsic Prothrombin Activation Pathway	*KLK7*
Retinol Biosynthesis	*LPL*
Triacylglycerol Degradation	*LPL*
GNRH Signaling	*MAP3K1*
Cellular Effects of Sildenafil (Viagra)	*MYLK*
4-1BB Signaling in T Lymphocytes	*NFKBIA*
Hypoxia Signaling in the Cardiovascular System	*NFKBIA*
ATM Signaling	*NFKBIA*
PTEN Signaling	*NGFR*
nNOS Signaling in Skeletal Muscle Cells	*NOS1*
nNOS Signaling in Neurons	*NOS1*
Dopamine-DARPP32 Feedback in cAMP Signaling	*NOS1*
Estrogen Receptor Signaling	*NR3C1*
Calcium-induced T Lymphocyte Apoptosis	*ORAI1*
BER pathway	*PARP1*
DNA Double-Strand Break Repair by Non-Homologous End Joining	*PARP1*
Role of Oct4 in Mammalian Embryonic Stem Cell Pluripotency	*PARP1*
UDP-N-acetyl-D-glucosamine Biosynthesis II	*PGM3*
GDP-glucose Biosynthesis	*PGM3*

Glucose and Glucose-1-phosphate Degradation	*PGM3*
UDP-N-acetyl-D-galactosamine Biosynthesis II	*PGM3*
Glycogen Degradation II	*PGM3*
Glycogen Degradation III	*PGM3*
Prostanoid Biosynthesis	*PTGS2*
Fatty Acid α-oxidation	*PTGS2*
Cancer Drug Resistance By Drug Efflux	*PTGS2*
GABA Receptor Signaling	*SLC6A12*
Sphingomyelin Metabolism	*SMPD1*
UVC-Induced MAPK Signaling	*SMPD1*
Mitotic Roles of Polo-Like Kinase	*TGFB1*
Cell Cycle: G1/S Checkpoint Regulation	*TGFB1*
Cyclins and Cell Cycle Regulation	*TGFB1*
Factors Promoting Cardiogenesis in Vertebrates	*TGFB1*
Wnt/β-catenin Signaling	*TGFB1*
Role of p14/p19ARF in Tumor Suppression	*TLR9*
Docosahexaenoic Acid (DHA) Signaling	*TLR9*
Apelin Pancreas Signaling Pathway	*TLR9*
Melanoma Signaling	*TLR9*
UVB-Induced MAPK Signaling	*TLR9*
Endometrial Cancer Signaling	*TLR9*
ErbB4 Signaling	*TLR9*
GDNF Family Ligand-Receptor Interactions	*TLR9*
Non-Small Cell Lung Cancer Signaling	*TLR9*
Estrogen-Dependent Breast Cancer Signaling	*TLR9*
Reelin Signaling in Neurons	*TLR9*
HER-2 Signaling in Breast Cancer	*TLR9*
TR/RXR Activation	*TLR9*
Melanocyte Development and Pigmentation Signaling	*TLR9*
ErbB Signaling	*TLR9*
FAK Signaling	*TLR9*
VEGF Signaling	*TLR9*
p53 Signaling	*TLR9*
Nitric Oxide Signaling in the Cardiovascular System	*TLR9*
Virus Entry via Endocytic Pathways	*TLR9*
Paxillin Signaling	*TLR9*

Glioma Signaling	*TLR9*
CXCR4 Signaling	*TLR9*
Aldosterone Signaling in Epithelial Cells	*TLR9*
CREB Signaling in Neurons	*TLR9*
mTOR Signaling	*TLR9*
EIF2 Signaling	*TLR9*
Hereditary Breast Cancer Signaling	*TLR9*
Regulation of eIF4 and p70S6K Signaling	*TLR9*
Breast Cancer Regulation by Stathmin1	*TLR9*
Glioblastoma Multiforme Signaling	*TLR9*
P2Y Purigenic Receptor Signaling Pathway	*TLR9*
Gap Junction Signaling	*TLR9*
Insulin Receptor Signaling	*TLR9*
Apelin Liver Signaling Pathway	*TNF*
IL-15 Production	*TYK2*
NOTE: By default, pathways were displayed with a –log (p-value) cutoff of 1.3, meaning that pathways with a p-value equal to or greater than 0.05 are hidden.	

Additional File 2: Table S2c. Ingenuity pathway analysis – top 3 pathways of atopy genes without 'bin' genes (n=108)

Pathways	-log (p-value)[1]	Ratio[2]	Molecules
Th1 and Th2 Activation Pathway	2,37E01	1,19E-01	*STAT6, IFNG, PTGDR2, IL12RB1, IL1RL1, TYK2, IL6R, IL12RB2, IL31, TBX21, IL13, TLR9, IL18, IL17RB, IL2, TGFB1, LTA, ICOS, IL9, S1PR1, CD86, IL4*
T Helper Cell Differentiation	2,18E01	2,19E-01	*IL21, IFNG, STAT6, IL12RB1, IL21R, IL6R, IL12RB2, TBX21, IL13, IL18, IL2, TGFB1, NGFR, ICOS, CD86, IL4*
Th2 Pathway	2,1E01	1,27E-01	*STAT6, IFNG, PTGDR2, IL12RB1, IL1RL1, TYK2, IL12RB2, IL31, TBX21, IL13, TLR9, IL17RB, IL2, TGFB1, ICOS, IL9, S1PR1, CD86, IL4*

[1]The –log (p-value) was calculated using a Fisher's exact test to determine the probability that the association between the atopy-related genes and the pathways is explained by a random chance alone. A –log (p-value) equal to or greater than 1.3, corresponding to a p-value of 0.05, was considered statistically significant.
[2]The ratio is calculated by the atopy-related genes found in each pathway divided by the total number of molecules in that pathway.

Additional File 2: Table S2d. Ingenuity pathway analysis – atopy genes without 'bin' genes (n=108)

Pathways	Molecules
Th1 and Th2 Activation Pathway	*STAT6, IFNG, PTGDR2, IL12RB1, IL1RL1, TYK2, IL6R, IL12RB2, IL31, TBX21, IL13, TLR9, IL18, IL17RB, IL2, TGFB1, LTA, ICOS, IL9, S1PR1, CD86, IL4*
T Helper Cell Differentiation	*IL21, IFNG, STAT6, IL12RB1, IL21R, IL6R, IL12RB2, TBX21, IL13, IL18, IL2, TGFB1, NGFR, ICOS, CD86, IL4*
Th2 Pathway	*STAT6, IFNG, PTGDR2, IL12RB1, IL1RL1, TYK2, IL12RB2, IL31, TBX21, IL13, TLR9, IL17RB, IL2, TGFB1, ICOS, IL9, S1PR1, CD86, IL4*
Neuroinflammation Signaling Pathway	*IFNG, NLRP3, BDNF, TYK2, IL6R, IRAK3, CCL5, TLR9, CSF1R, TLR2, PLA2G4A, IL18, CCL2, TGFB1, TLR6, CD86, NOS2, MMP9, SLC6A12, IL4*
TREM1 Signaling	*TLR2, IL18, NOD2, NLRP3, CCL2, IL1RL1, TLR6, NOD1, CD86, FCGR2B, TLR9, CCL7*
Role of Pattern Recognition Receptors in Recognition of Bacteria and Viruses	*IFNG, NLRP3, CCL5, TLR9, IL13, TLR2, IL18, NOD2, TGFB1, IL2, LTA, TLR6, NOD1, IL4*
Th1 Pathway	*IFNG, IL18, IL2, IL12RB1, LTA, ICOS, TYK2, IL6R, CD86, IL12RB2, TBX21, TLR9, IL4*
Altered T Cell and B Cell Signaling in Rheumatoid Arthritis	*TLR2, IL21, IFNG, IL18, IL2, TGFB1, LTA, TLR6, CD86, TLR9, IL4*
Atherosclerosis Signaling	*IFNG, PLA2G4A, IL18, SELP, CCL2, TGFB1, SERPINA1, CCL11, ALOX5, PLA2G7, MMP9*
HMGB1 Signaling	*IFNG, IL18, CCL2, IL2, TGFB1, NGFR, LTA, TLR9, IL13, SERPINE1, IL4*
Granulocyte Adhesion and Diapedesis	*IL18, SELP, CCL2, IL1RL1, NGFR, CCL26, CCL5, MMP12, HRH4, CCL11, MMP9, CCL7*
Hepatic Fibrosis / Hepatic Stellate Cell Activation	*IFNG, CCL2, IL1RL1, TGFB1, NGFR, IL6R, CD14, COL6A5, CCL5, SERPINE1, MMP9, IL4*
IL-12 Signaling and Production in Macrophages	*TLR2, IFNG, STAT6, IL18, TGFB1, IL12RB1, IL12RB2, SERPINA1, NOS2, TLR9, IL4*
Hepatic Cholestasis	*IFNG, IL18, IL1RL1, IL2, TGFB1, NGFR, LTA, CD14, IRAK3, IL13, IL4*
LXR/RXR Activation	*IL18, CCL2, IL1RL1, NGFR, CD14, SERPINA1, GC, NOS2, MMP9, CCL7*
Role of Macrophages, Fibroblasts and Endothelial Cells in Rheumatoid Arthritis	*IL1RL1, IL6R, CCL5, IRAK3, TLR9, TLR2, IL18, TRAF3IP2, CCL2, TGFB1, LTA, NGFR, TLR6, NOS2*
Th17 Activation Pathway	*IL21, IFNG, IL12RB1, IL21R, IL6R, TYK2, IL12RB2, PTGER2, IRAK3*
Communication between Innate and Adaptive Immune Cells	*TLR2, IFNG, IL18, IL2, TLR6, CD86, CCL5, TLR9, IL4*
Eicosanoid Signaling	*PLA2G4A, PTGDR, TBXA2R, CYSLTR1, PTGER2, ALOX5, PLA2G7, CYSLTR2*
Role of JAK1 and JAK3 in γc Cytokine Signaling	*IL7R, IL21, STAT6, IL2, IL21R, IL9, TLR9, IL4*
Role of Cytokines in Mediating Communication between Immune Cells	*IL21, IFNG, IL18, IL2, TGFB1, IL13, IL4*

Glucocorticoid Receptor Signaling	*IFNG, CCL5, CCL11, IL13, TLR9, SCGB1A1, CCL2, TGFB1, IL2, SERPINE1, NOS2, ADRB2, IL4*
Colorectal Cancer Metastasis Signaling	*TLR2, IFNG, TGFB1, IL6R, TLR6, TYK2, PTGER2, NOS2, MMP12, TLR9, MMP9*
Differential Regulation of Cytokine Production in Intestinal Epithelial Cells by IL-17A and IL-17F	*IFNG, CCL2, IL9, CCL5, IL13*
Toll-like Receptor Signaling	*TLR2, IL18, IL1RL1, TLR6, CD14, IRAK3, TLR9*
Crosstalk between Dendritic Cells and Natural Killer Cells	*IFNG, IL18, IL2, LTA, CD86, TLR9, IL4*
Agranulocyte Adhesion and Diapedesis	*IL18, SELP, CCL2, CCL26, CCL5, MMP12, CCL11, MMP9, CCL7*
Production of Nitric Oxide and Reactive Oxygen Species in Macrophages	*TLR2, IFNG, NGFR, CAT, TYK2, SERPINA1, NOS2, TLR9, IL4*
MSP-RON Signaling Pathway	*TLR2, IFNG, KLK7, CCL2, NOS2, TLR9*
Differential Regulation of Cytokine Production in Macrophages and T Helper Cells by IL-17A and IL-17F	*CCL2, IL9, CCL5, IL13*
Role of Hypercytokinemia/hyperchemokinemia in the Pathogenesis of Influenza	*IFNG, IL18, CCL2, IL9, CCL5*
iNOS Signaling	*IFNG, TYK2, CD14, IRAK3, NOS2*
Fc Epsilon RI Signaling	*BTK, PLA2G4A, FCER1A, MS4A2, IL13, TLR9, IL4*
Role of NFAT in Regulation of the Immune Response	*BTK, FCER1A, MS4A2, CD86, TLR9, FCGR2B, ORAI1, ITK*
LPS/IL-1 Mediated Inhibition of RXR Function	*IL18, IL1RL1, NGFR, CAT, CD14, PPARGC1B, GSTO2, GSTP1*
Type I Diabetes Mellitus Signaling	*IFNG, IL2, NGFR, LTA, CD86, NOS2*
Role of Osteoblasts, Osteoclasts and Chondrocytes in Rheumatoid Arthritis	*IFNG, IL18, TGFB1, IL1RL1, NGFR, TLR9, CSF1R, IL4*
Tec Kinase Signaling	*BTK, STAT6, TYK2, FCER1A, MS4A2, TLR9, ITK*
IL-10 Signaling	*IL18, IL1RL1, TYK2, CD14, FCGR2B*
Role of IL-17A in Arthritis	*CCL2, CCL5, NOS2, TLR9, CCL7*
NF-κB Signaling	*TLR2, IL18, NGFR, LTA, TLR6, IRAK3, TLR9*
VDR/RXR Activation	*IFNG, IL1RL1, IL2, CD14, CCL5*
Role of MAPK Signaling in the Pathogenesis of Influenza	*IFNG, PLA2G4A, CCL2, CCL5, PLA2G7*
CD28 Signaling in T Helper Cells	*ARPC1B, IL2, CD86, TLR9, CTLA4, ITK*
Dendritic Cell Maturation	*TLR2, IL18, NGFR, LTA, CD86, FCGR2B, TLR9*
IL-6 Signaling	*IL18, IL1RL1, NGFR, IL6R, CD14, TLR9*
IL-17 Signaling	*TRAF3IP2, CCL2, NOS2, TLR9, CCL11*
Graft-versus-Host Disease Signaling	*IFNG, IL18, IL2, CD86*
Inflammasome pathway	*IL18, NOD2, NLRP3*
Endocannabinoid Cancer Inhibition Pathway	*NOS1, TWIST1, SMPD1, TRPV1, NOS2, TLR9*
Airway Inflammation in Asthma	*IL13, IL4*

STAT3 Pathway	*IL17RB, TGFB1, NGFR, TYK2, IL9*
Citrulline-Nitric Oxide Cycle	*NOS1, NOS2*
T Cell Receptor Signaling	*BTK, PAG1, TLR9, CTLA4, ITK*
p38 MAPK Signaling	*PLA2G4A, IL18, IL1RL1, TGFB1, IRAK3*
HIF1α Signaling	*NOS1, NOS2, MMP12, TLR9, MMP9*
Endothelin-1 Signaling	*NOS1, PLA2G4A, PTGER2, NOS2, TLR9, PLA2G7*
Phagosome Formation	*TLR2, TLR6, FCER1A, FCGR2B, TLR9*
CCR3 Signaling in Eosinophils	*PLA2G4A, MYLK, CCL26, TLR9, CCL11*
Chemokine Signaling	*CCL2, CCL5, CCL11, CCL7*
IL-17A Signaling in Airway Cells	*TRAF3IP2, TYK2, CCL11, TLR9*
Leukocyte Extravasation Signaling	*BTK, CTNNA3, MMP12, TLR9, MMP9, ITK*
Osteoarthritis Pathway	*TLR2, IL1RL1, TGFB1, NOS2, MMP12, MMP9*
IL-17A Signaling in Fibroblasts	*TRAF3IP2, CCL2, CCL7*
IL-15 Signaling	*STAT6, TYK2, TLR9, IL4*
Allograft Rejection Signaling	*IFNG, IL2, CD86, IL4*
Systemic Lupus Erythematosus Signaling	*IL18, IL2, IL6R, CD86, FCGR2B, TLR9*
IL-4 Signaling	*STAT6, TYK2, TLR9, IL4*
MIF Regulation of Innate Immunity	*PLA2G4A, CD14, NOS2*
Ceramide Signaling	*NGFR, S1PR1, SMPD1, TLR9*
Role of IL-17F in Allergic Inflammatory Airway Diseases	*TRAF3IP2, CCL2, CCL7*
Hematopoiesis from Multipotent Stem Cells	*IL2, IL4*
Acute Phase Response Signaling	*IL18, NGFR, IL6R, SERPINA1, SERPINE1*
Autoimmune Thyroid Disease Signaling	*IL2, CD86, IL4*
Hematopoiesis from Pluripotent Stem Cells	*IL2, IL9, IL4*
Primary Immunodeficiency Signaling	*IL7R, BTK, ICOS*
Apelin Cardiomyocyte Signaling Pathway	*TGFB1, CAT, MYLK, TLR9*
Superpathway of Citrulline Metabolism	*NOS1, NOS2*
G-Protein Coupled Receptor Signaling	*PTGDR, TBXA2R, S1PR1, PTGER2, TLR9, ADRB2*
Regulation of the Epithelial-Mesenchymal Transition Pathway	*TGFB1, TYK2, TWIST1, TLR9, MMP9*
Pancreatic Adenocarcinoma Signaling	*TGFB1, TYK2, TLR9, MMP9*
iCOS-iCOSL Signaling in T Helper Cells	*IL2, ICOS, TLR9, ITK*
SPINK1 Pancreatic Cancer Pathway	*KLK7, TGFB1, MMP12*
Renin-Angiotensin Signaling	*CCL2, CCL5, PTGER2, TLR9*
Apelin Cardiac Fibroblast Signaling Pathway	*TGFB1, SERPINE1*
cAMP-mediated signaling	*PTGDR, TBXA2R, S1PR1, PTGER2, ADRB2*
Human Embryonic Stem Cell Pluripotency	*BDNF, TGFB1, S1PR1, TLR9*

Role of JAK1, JAK2 and TYK2 in Interferon Signaling	*IFNG, TYK2*
Role of JAK family kinases in IL-6-type Cytokine Signaling	*IL6R, TYK2*
Role of PI3K/AKT Signaling in the Pathogenesis of Influenza	*IFNG, CCL5, TLR9*
SPINK1 General Cancer Pathway	*IL6R, TYK2, TLR9*
Antiproliferative Role of TOB in T Cell Signaling	*IL2, TGFB1*
Neurotrophin/TRK Signaling	*BDNF, NGFR, TLR9*
FcγRIIB Signaling in B Lymphocytes	*BTK, FCGR2B, TLR9*
Macropinocytosis Signaling	*CD14, TLR9, CSF1R*
JAK/Stat Signaling	*STAT6, TYK2, TLR9*
IL-7 Signaling Pathway	*IL7R, IFNG, TLR9*
Glutathione-mediated Detoxification	*GSTO2, GSTP1*
Synaptic Long Term Depression	*NOS1, PLA2G4A, NOS2, PLA2G7*
Inhibition of Angiogenesis by TSP1	*TGFB1, MMP9*
Coagulation System	*SERPINA1, SERPINE1*
MIF-mediated Glucocorticoid Regulation	*PLA2G4A, CD14*
CTLA4 Signaling in Cytotoxic T Lymphocytes	*CD86, TLR9, CTLA4*
B Cell Development	*IL7R, CD86*
Interferon Signaling	*IFNG, TYK2*
PPAR Signaling	*IL18, IL1RL1, NGFR*
B Cell Receptor Signaling	*BTK, PAG1, FCGR2B, TLR9*
Inhibition of Matrix Metalloproteases	*MMP12, MMP9*
Xenobiotic Metabolism Signaling	*CAT, GSTO2, NOS2, TLR9, GSTP1*
Antioxidant Action of Vitamin C	*PLA2G4A, GSTO2, PLA2G7*
Gαs Signaling	*PTGDR, PTGER2, ADRB2*
NRF2-mediated Oxidative Stress Response	*CAT, GSTO2, TLR9, GSTP1*
Adrenomedullin signaling pathway	*IL18, LTA, MYLK, TLR9*
Amyotrophic Lateral Sclerosis Signaling	*NOS1, CAT, TLR9*
UVA-Induced MAPK Signaling	*SMPD1, TLR9, PARP1*
IL-8 Signaling	*IL9, IRAK3, TLR9, MMP9*
IL-9 Signaling	*IL9, TLR9*
Neuroprotective Role of THOP1 in Alzheimer's Disease	*IFNG, KLK7, MMP9*
NGF Signaling	*NGFR, SMPD1, TLR9*
Sphingosine-1-phosphate Signaling	*S1PR1, SMPD1, TLR9*
FXR/RXR Activation	*IL18, SERPINA1, GC*
Actin Cytoskeleton Signaling	*ARPC1B, CD14, MYLK, TLR9*
GP6 Signaling Pathway	*BTK, COL6A5, TLR9*
PI3K Signaling in B	*BTK, FCGR2B, IL4*

Lymphocytes	
p70S6K Signaling	*BTK, TLR9, IL4*
Corticotropin Releasing Hormone Signaling	*NOS1, BDNF, NOS2*
Cardiac Hypertrophy Signaling	*TGFB1, IL6R, TLR9, ADRB2*
Gα12/13 Signaling	*BTK, TBXA2R, TLR9*
Aryl Hydrocarbon Receptor Signaling	*TGFB1, GSTO2, GSTP1*
Phospholipase C Signaling	*BTK, PLA2G4A, FCGR2B, ITK*
Ephrin A Signaling	*NGFR, TLR9*
Retinoic acid Mediated Apoptosis Signaling	*IFNG, PARP1*
Phospholipases	*PLA2G4A, PLA2G7*
Type II Diabetes Mellitus Signaling	*NGFR, SMPD1, TLR9*
Lymphotoxin β Receptor Signaling	*LTA, TLR9*
Relaxin Signaling	*NOS2, TLR9, MMP9*
CNTF Signaling	*TYK2, TLR9*
IL-2 Signaling	*IL2, TLR9*
PKCθ Signaling in T Lymphocytes	*IL2, CD86, TLR9*
Tight Junction Signaling	*TGFB1, NGFR, MYLK*
Cdc42 Signaling	*ARPC1B, MYLK, ITK*
ErbB2-ErbB3 Signaling	*TYK2, TLR9*
Glioma Invasiveness Signaling	*TLR9, MMP9*
CD40 Signaling	*LTA, TLR9*
Apelin Adipocyte Signaling Pathway	*CAT, GSTP1*
Antiproliferative Role of Somatostatin Receptor 2	*NOS1, TLR9*
Small Cell Lung Cancer Signaling	*NOS1, TLR9*
Regulation of IL-2 Expression in Activated and Anergic T Lymphocytes	*IL2, TGFB1*
Renal Cell Carcinoma Signaling	*TGFB1, TLR9*
IL-3 Signaling	*STAT6, TLR9*
Regulation of Actin-based Motility by Rho	*ARPC1B, MYLK*
ILK Signaling	*NOS2, TLR9, MMP9*
Axonal Guidance Signaling	*ARPC1B, BDNF, NGFR, TLR9, MMP9*
LPS-stimulated MAPK Signaling	*CD14, TLR9*
FLT3 Signaling in Hematopoietic Progenitor Cells	*STAT6, TLR9*
PEDF Signaling	*BDNF, TLR9*
TGF-β Signaling	*TGFB1, SERPINE1*
3-phosphoinositide Biosynthesis	*ICOS, CD86, TLR9*
Bladder Cancer Signaling	*MMP12, MMP9*
VEGF Family Ligand-Receptor Interactions	*PLA2G4A, TLR9*
PDGF Signaling	*TYK2, TLR9*

Clathrin-mediated Endocytosis Signaling	*ARPC1B, SERPINA1, TLR9*
Acute Myeloid Leukemia Signaling	*TLR9, CSF1R*
AMPK Signaling	*CFTR, TLR9, ADRB2*
Prostate Cancer Signaling	*TLR9, GSTP1*
Integrin Signaling	*ARPC1B, MYLK, TLR9*
CDK5 Signaling	*BDNF, NGFR*
PAK Signaling	*MYLK, TLR9*
Chronic Myeloid Leukemia Signaling	*TGFB1, TLR9*
Mouse Embryonic Stem Cell Pluripotency	*TYK2, TLR9*
Neuropathic Pain Signaling In Dorsal Horn Neurons	*BDNF, TLR9*
Superpathway of Inositol Phosphate Compounds	*ICOS, CD86, TLR9*
Telomerase Signaling	*IL2, TLR9*
Rac Signaling	*ARPC1B, TLR9*
Gαi Signaling	*TBXA2R, S1PR1*
RhoA Signaling	*ARPC1B, MYLK*
Apelin Endothelial Signaling Pathway	*CCL2, TLR9*
Signaling by Rho Family GTPases	*ARPC1B, MYLK, TLR9*
Sperm Motility	*PLA2G4A, PLA2G7*
Role of NANOG in Mammalian Embryonic Stem Cell Pluripotency	*TYK2, TLR9*
fMLP Signaling in Neutrophils	*ARPC1B, TLR9*
Iron homeostasis signaling pathway	*IL6R, TYK2*
Ovarian Cancer Signaling	*TLR9, MMP9*
Gαq Signaling	*BTK, TLR9*
Germ Cell-Sertoli Cell Junction Signaling	*TGFB1, TLR9*
Sertoli Cell-Sertoli Cell Junction Signaling	*NOS1, NOS2*
PPARα/RXRα Activation	*IL1RL1, TGFB1*
RAR Activation	*TGFB1, PARP1*
ERK/MAPK Signaling	*PLA2G4A, TLR9*
Thrombin Signaling	*MYLK, TLR9*
Role of NFAT in Cardiac Hypertrophy	*TGFB1, TLR9*
Molecular Mechanisms of Cancer	*TGFB1, TYK2, TLR9*
Protein Kinase A Signaling	*TGFB1, NGFR, MYLK*
Huntington's Disease Signaling	*BDNF, TLR9*
Sirtuin Signaling Pathway	*NOS2, PARP1*
Circadian Rhythm Signaling	*ADCYAP1R1*
GPCR-Mediated Integration of Enteroendocrine Signaling Exemplified by an L Cell	*ADRB2*

Leukotriene Biosynthesis	*ALOX5*
Actin Nucleation by ARP-WASP Complex	*ARPC1B*
Remodeling of Epithelial Adherens Junctions	*ARPC1B*
Fcγ Receptor-mediated Phagocytosis in Macrophages and Monocytes	*ARPC1B*
Epithelial Adherens Junction Signaling	*ARPC1B*
RhoGDI Signaling	*ARPC1B*
Ephrin Receptor Signaling	*ARPC1B*
Thyroid Cancer Signaling	*BDNF*
G Beta Gamma Signaling	*BTK*
Superoxide Radicals Degradation	*CAT*
Ethanol Degradation IV	*CAT*
Mitochondrial Dysfunction	*CAT*
Pathogenesis of Multiple Sclerosis	*CCL5*
IL-17A Signaling in Gastric Cells	*CCL5*
CCR5 Signaling in Macrophages	*CCL5*
Nur77 Signaling in T Lymphocytes	*CD86*
Catecholamine Biosynthesis	*DBH*
HIPPO signaling	*FRMD6*
Ascorbate Recycling (Cytosolic)	*GSTO2*
Vitamin-C Transport	*GSTO2*
Glutathione Redox Reactions I	*GSTP1*
Histamine Degradation	*HNMT*
Role of Lipids/Lipid Rafts in the Pathogenesis of Influenza	*IFNG*
Antigen Presentation Pathway	*IFNG*
Role of PKR in Interferon Induction and Antiviral Response	*IFNG*
Role of Wnt/GSK-3β Signaling in the Pathogenesis of Influenza	*IFNG*
Role of BRCA1 in DNA Damage Response	*IFNG*
Protein Ubiquitination Pathway	*IFNG*
Cholecystokinin/Gastrin-mediated Signaling	*IL18*
OX40 Signaling Pathway	*IL2*
3-phosphoinositide Degradation	*INPP4A*
IL-1 Signaling	*IRAK3*
Intrinsic Prothrombin Activation Pathway	*KLK7*
TNFR2 Signaling	*LTA*
Activation of IRF by Cytosolic Pattern Recognition Receptors	*LTA*
Airway Pathology in Chronic Obstructive Pulmonary Disease	*MMP9*
Cellular Effects of Sildenafil	*MYLK*

(Viagra)	
Induction of Apoptosis by HIV1	*NGFR*
PTEN Signaling	*NGFR*
nNOS Signaling in Skeletal Muscle Cells	*NOS1*
nNOS Signaling in Neurons	*NOS1*
Phagosome Maturation	*NOS1*
Dopamine-DARPP32 Feedback in cAMP Signaling	*NOS1*
Opioid Signaling Pathway	*NOS1*
Calcium-induced T Lymphocyte Apoptosis	*ORAI1*
BER pathway	*PARP1*
DNA Double-Strand Break Repair by Non-Homologous End Joining	*PARP1*
Granzyme B Signaling	*PARP1*
Role of Oct4 in Mammalian Embryonic Stem Cell Pluripotency	*PARP1*
Death Receptor Signaling	*PARP1*
Apoptosis Signaling	*PARP1*
UDP-N-acetyl-D-glucosamine Biosynthesis II	*PGM3*
GDP-glucose Biosynthesis	*PGM3*
Glucose and Glucose-1-phosphate Degradation	*PGM3*
UDP-N-acetyl-D-galactosamine Biosynthesis II	*PGM3*
Glycogen Degradation II	*PGM3*
Glycogen Degradation III	*PGM3*
GABA Receptor Signaling	*SLC6A12*
Sphingomyelin Metabolism	*SMPD1*
UVC-Induced MAPK Signaling	*SMPD1*
Mitotic Roles of Polo-Like Kinase	*TGFB1*
Cell Cycle: G1/S Checkpoint Regulation	*TGFB1*
Cyclins and Cell Cycle Regulation	*TGFB1*
Factors Promoting Cardiogenesis in Vertebrates	*TGFB1*
Adipogenesis pathway	*TGFB1*
Wnt/β-catenin Signaling	*TGFB1*
Role of p14/p19ARF in Tumor Suppression	*TLR9*
Docosahexaenoic Acid (DHA) Signaling	*TLR9*
Apelin Pancreas Signaling Pathway	*TLR9*
Melanoma Signaling	*TLR9*
UVB-Induced MAPK Signaling	*TLR9*

EGF Signaling	*TLR9*
Endometrial Cancer Signaling	*TLR9*
Thrombopoietin Signaling	*TLR9*
Myc Mediated Apoptosis Signaling	*TLR9*
ErbB4 Signaling	*TLR9*
GM-CSF Signaling	*TLR9*
GDNF Family Ligand-Receptor Interactions	*TLR9*
Non-Small Cell Lung Cancer Signaling	*TLR9*
Angiopoietin Signaling	*TLR9*
Growth Hormone Signaling	*TLR9*
Estrogen-Dependent Breast Cancer Signaling	*TLR9*
Leptin Signaling in Obesity	*TLR9*
Erythropoietin Signaling	*TLR9*
Prolactin Signaling	*TLR9*
FGF Signaling	*TLR9*
Reelin Signaling in Neurons	*TLR9*
NF-κB Activation by Viruses	*TLR9*
HER-2 Signaling in Breast Cancer	*TLR9*
TR/RXR Activation	*TLR9*
RANK Signaling in Osteoclasts	*TLR9*
Melanocyte Development and Pigmentation Signaling	*TLR9*
ErbB Signaling	*TLR9*
FAK Signaling	*TLR9*
VEGF Signaling	*TLR9*
SAPK/JNK Signaling	*TLR9*
p53 Signaling	*TLR9*
IGF-1 Signaling	*TLR9*
Nitric Oxide Signaling in the Cardiovascular System	*TLR9*
Virus Entry via Endocytic Pathways	*TLR9*
HGF Signaling	*TLR9*
Paxillin Signaling	*TLR9*
Glioma Signaling	*TLR9*
Endocannabinoid Developing Neuron Pathway	*TLR9*
Natural Killer Cell Signaling	*TLR9*
Role of Tissue Factor in Cancer	*TLR9*
14-3-3-mediated Signaling	*TLR9*
P2Y Purigenic Receptor Signaling Pathway	*TLR9*
Insulin Receptor Signaling	*TLR9*
Hereditary Breast Cancer Signaling	*TLR9*
Regulation of eIF4 and p70S6K Signaling	*TLR9*

Aldosterone Signaling in Epithelial Cells	*TLR9*
Glioblastoma Multiforme Signaling	*TLR9*
CXCR4 Signaling	*TLR9*
eNOS Signaling	*TLR9*
Gap Junction Signaling	*TLR9*
CREB Signaling in Neurons	*TLR9*
mTOR Signaling	*TLR9*
EIF2 Signaling	*TLR9*
Breast Cancer Regulation by Stathmin1	*TLR9*
IL-22 Signaling	*TYK2*
IL-15 Production	*TYK2*
Role of JAK2 in Hormone-like Cytokine Signaling	*TYK2*
Oncostatin M Signaling	*TYK2*
PI3K/AKT Signaling	*TYK2*
NOTE: By default, pathways were displayed with a –log (p-value) cutoff of 1.3, meaning that pathways with a p-value equal to or greater than 0.05 are hidden.	
